# Testing the Ortholog Conjecture with Comparative Functional Genomic Data from Mammals

**DOI:** 10.1371/journal.pcbi.1002073

**Published:** 2011-06-09

**Authors:** Nathan L. Nehrt, Wyatt T. Clark, Predrag Radivojac, Matthew W. Hahn

**Affiliations:** 1 School of Informatics and Computing, Indiana University, Bloomington, Indiana, United States of America; 2 Department of Biology, Indiana University, Bloomington, Indiana, United States of America; University of Chicago, United States of America

## Abstract

A common assumption in comparative genomics is that orthologous genes share greater functional similarity than do paralogous genes (the “ortholog conjecture”). Many methods used to computationally predict protein function are based on this assumption, even though it is largely untested. Here we present the first large-scale test of the ortholog conjecture using comparative functional genomic data from human and mouse. We use the experimentally derived functions of more than 8,900 genes, as well as an independent microarray dataset, to directly assess our ability to predict function using both orthologs and paralogs. Both datasets show that paralogs are often a much better predictor of function than are orthologs, even at lower sequence identities. Among paralogs, those found within the same species are consistently more functionally similar than those found in a different species. We also find that paralogous pairs residing on the same chromosome are more functionally similar than those on different chromosomes, perhaps due to higher levels of interlocus gene conversion between these pairs. In addition to offering implications for the computational prediction of protein function, our results shed light on the relationship between sequence divergence and functional divergence. We conclude that the most important factor in the evolution of function is not amino acid sequence, but rather the cellular context in which proteins act.

## Author Summary

The use of model organisms in biological research rests upon the assumption that gene and protein functions discovered in one organism are likely to be the same or similar in another organism. Hence, the assumption that experiments in mouse will tell us about the function of genes in humans. A guiding principle in the assignment of function from one organism to another is that single-copy genes (“orthologs”) are statistically more likely to provide functional information than are multi-copy genes, whether in the same organism or different organisms. Here we have tested this idea by examining genes with known functions in human and mouse. Surprisingly, we find that multi-copy genes are equally or more likely to provide accurate functional information than are single-copy genes. Our results suggest that the organism itself plays at least as large a role in determining the function of genes as does the particular sequence of the gene alone. This insight will benefit the assignment of function to genes whose roles are not yet known by widening the pool of appropriate genes from which function can be inferred.

## Introduction

The potential for gene duplication to generate evolutionary novelty was first noted in 1918 by Calvin Bridges (cited in [1]), and the idea quickly found many supporters [2]–[4]. The advent of protein-sequencing technologies in the 1950s and ‘60s confirmed the presence of many gene duplicates, and once again researchers championed the importance of duplication in evolution [5]. Today, the sequencing of hundreds of whole genomes has revealed the ubiquity of gene duplicates in all domains of life, and a growing number of empirical and computational studies have provided direct evidence for the role of gene duplication in adaptation [6].

As the first protein-sequence data became available, Zuckerkandl and Pauling [7] made the distinction between “duplication-independent homology” and “duplication-dependent homology,” what we now refer to as orthology and paralogy, respectively [8], [9]. They recognized that the paralogous α-, β-, and γ-hemoglobin chains present in all jawed vertebrates were less functionally similar to each other than were orthologous copies between closely related species, largely because they had been diverged for a very long period of time. Despite the fact that this and a small handful of other examples were confined to cases with very deep divergences between paralogs, the idea that orthologs were more similar in function than paralogs continued to be a basic tenet of comparative studies. As the first large genome sequencing projects were completed and thousands of previously unknown genes had to be annotated, this idea re-appeared in the seminal papers of the field now known as phylogenomics: “Normally, orthologs retain the same function in the course of evolution, whereas paralogs evolve new functions, even if related to the original one. Thus, identification of orthologs is critical for reliable prediction of gene functions in newly sequenced genomes” [10]. Similar statements can be found in many papers (e.g. [11]–[18]), and—as pointed out by Studer and Robinson-Rechavi [19]—can even be found in the primer on phylogenetics at the National Center for Biotechnology Information (NCBI) website (http://www.ncbi.nlm.nih.gov/About/primer/phylo.html).

We refer to the hypothesis that orthologs are more likely to be functionally similar than are paralogs as the “ortholog conjecture” (cf. [15]). In fact, only rarely has it even been noted that this idea is a *hypothesis* about functional similarity [15], [19]—in most studies it is either assumed to be true or is supported by evidence from a small number of genes. It is certainly the case that increased rates of sequence evolution often follow gene duplication [20]–[23], but rarely are these changes connected to functional differences (e.g. [24]). Moreover, one of the three major hypotheses for the maintenance of gene duplicates (subfunctionalization) does not require any functional change, and another (gene dosage) even prohibits such changes from occurring [6]. There have been studies comparing rates of adaptive evolution in duplicates versus single-copy genes, but these have provided conflicting results [25], [26]; rates of adaptive evolution may also be poor predictors of overall functional similarity [27]. We do not know of any study that has systematically tested the ortholog conjecture.

A large number of methods have been developed to identify orthologous relationships among proteins. These methods range from simple pairwise comparisons, to standard phylogenetic tree-building, to probabilistic assignment using Bayesian analyses [28]–[30]. Several databases provide predicted orthologs [31]–[33], and whole scientific meetings are devoted to their study [34]. While the identification of orthologs is certainly highly relevant to many evolutionary questions—especially in systematics—many of these methods are explicitly made for functional inference. Note also that in most cases these methods are only distinguishing between orthologs and outparalogs [35]: that is, between an ortholog and a paralog that duplicated before the speciation event separating the orthologs, and that is therefore almost always more diverged at the sequence level (Figure S1). Paralogs more closely related to each other than either is to an ortholog (“inparalogs”; Figure S1) are by definition co-orthologous to a single-copy gene from another species, and neither represents the “true” ortholog (though see [36] for more complex sets of relationships between inparalogs).

In this paper we directly test the ortholog conjecture using comparative functional genomic data. We use experimentally derived functional assignments of more than 8,900 genes from mouse and human, as well as a microarray dataset that includes 25 tissues in both mouse and human, to directly assess our ability to predict function using orthologs and paralogs. We use this pair of species both because they are two of the best-studied and best-annotated organisms and because homologous relationships are easy to identify due to their relatively recent divergence time. Because paralogs are almost always either more- or less-related to a focal gene than an ortholog (for inparalogs or outparalogs, respectively), it is meaningless to compare the predictive power of all orthologs to all paralogs; it seems obvious that closely related orthologs will be more similar in function than distantly related paralogs, and vice versa. Instead, we focus on the predictive power of both orthologs and paralogs as a function of protein sequence divergence. Our results demonstrate that paralogous genes from the same species are often a much better predictor of functional divergence than are orthologs or paralogs from different species, even at lower sequence identities.

## Results

### Functional similarity between orthologs and all paralogs

**Figure 1:**
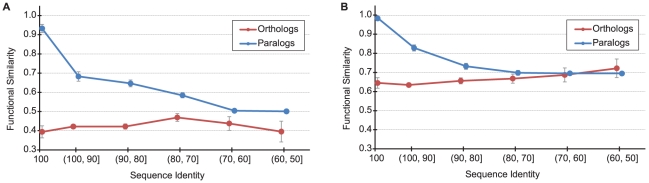
The relationship between functional similarity and sequence identity for human-mouse orthologs (red) and all paralogs (blue). Standard error bars are shown. (**A**) Biological Process ontology, (**B**) Molecular Function ontology.

Functional similarity was calculated between all pairs of homologous proteins (i.e. those in the same gene family) in human and mouse for which there is experimentally defined function for both members of the pair. These pairs include 2,579 one-to-one orthologs between human and mouse and 21,771 paralogous comparisons of any type. The experiments used to annotate these genes come from 12,204 unique published papers whose results are collected in the Gene Ontology (GO) database; in a later section we carry out an independent analysis using microarray data to measure functional similarity. Figure 1 shows the relationship between experimentally defined functional similarity and protein sequence identity for both orthologous and paralogous pairs.

Functional similarity can be measured for both the Biological Process and Molecular Function categories defined in the GO database. For the Biological Process category, Figure 1A shows that the average functional similarity between human-mouse orthologs was consistently between 0.4 and 0.5 over the entire range of sequence identities. Similarly, for the Molecular Function category, Figure 1B shows functional similarity was between 0.6 and 0.7 over the entire range. The relatively low levels of similarity are at least partially influenced by the sparsity of annotation, but this is unlikely to affect comparisons between classes of homologs. Most strikingly, there is no correlation between functional similarity and protein sequence identity for orthologs (Figure 1): two orthologs have the same average functional similarity at 99% as they do at 51% (see Discussion). This relationship holds no matter the exact measure of sequence identity used; there is also no relationship observed between selective constraint (i.e. *d*_N_/*d*_S_) and functional similarity (Figure S2). In contrast, the functional similarity between paralogs is positively correlated with sequence identity for both ontologies, but shows a steeper decline for Biological Process. In Figure 1, the protein pairs included in the paralog category consist of both inparalogs and outparalogs; thus, the distributions largely consist of inparalogs in the high sequence identity ranges and outparalogs in the low sequence identity ranges.

Contrary to a common assumption (the “ortholog conjecture”), the functional similarity between paralogs is significantly higher than that between orthologs for high sequence identities (≥70% for Biological Process; *P*<10^−5^; ≥80% for Molecular Function, *P*<10^−4^;Wilcoxon test) and functional similarity is nearly the same for the different types of homologs as sequence identity approaches 50%. The curves do not provide comparable information for sequence identities below 50% because of an insufficient number of 1-to-1 orthologous pairs with very low identity.

### Functional similarity between orthologs and subtypes of paralogs

**Figure 2:**
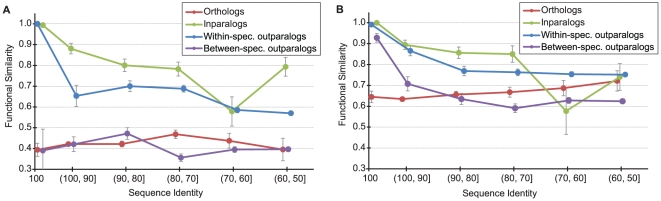
The relationship between functional similarity and sequence identity for human-mouse orthologs (red), inparalogs (green), within-species outparalogs (blue), between-species outparalogs (purple). Standard error bars are shown. (**A**) Biological Process ontology, (**B**) Molecular Function ontology.

While the ortholog data can be easily understood from Figure 1, the combination of several types of paralogs obscures the interpretation of the functional similarity between paralogs. We therefore separated paralogs into three further classes: (i) inparalogs, (ii) within-species outparalogs, and (iii) between-species outparalogs (Figure S1). Inparalogs and within-species outparalogs include protein pairs from the same species (human-human or mouse-mouse) whereas the between-species outparalogs include human-mouse pairs only. Figure 2 presents results for these separate types of paralogs; note that the curves for orthologs are identical to those in Figure 1. In total, there were 597 inparalogous pairs compared, 11,334 within-species outparalogs, and 9,840 between-species outparalogs.

The functional similarity curves show a clear difference between subtypes of paralogs. Inparalogs appear to be most functionally similar to one another, and their functional similarity is positively correlated with sequence identity in both ontologies. Within-species outparalogs have a slightly steeper decline than inparalogs, but are significantly more functionally similar than either between-species outparalogs or orthologs. The between-species outparalogs show trends most similar to orthologs. In fact, in the Biological Process category, these two curves are nearly identical. However, in the Molecular Function category, the more sequence-similar outparalogs have slightly higher functional similarity than do orthologs, while the less-similar outparalogs have lower functional similarity than do orthologs. In the Discussion we propose an explanation for these relationships.

### Family-based analyses

In addition to a large-scale view of functional similarity, it is also useful to take a family-based view in order to compare the predictive power of paralogs and orthologs within the same family. We asked, for a given family, first whether an ortholog or a paralog was more similar at the sequence level, and then whether an ortholog or the particular paralog was more similar at the functional level.

The counts for the groups were obtained as follows: for each family, only one target protein (functionally annotated) was selected uniformly randomly from all proteins with at least one ortholog and at least one paralog in the family, and all its functionally annotated homologs were collected. We then asked whether at least one of the paralogs had higher sequence similarity than the ortholog, and then whether it had higher or lower functional similarity. This analysis required functionally annotated triples within gene families (i.e. the target gene, an ortholog, and a paralog of any type); thus 1-to-many and many-to-many orthologous relationships were included in this analysis. In cases where multiple genes were co-orthologous to the target, the ortholog having the highest sequence identity with the selected target protein was used for comparison. Note that each gene family was counted only once in this analysis, preventing families with large numbers of lineage-specific duplications from biasing the results. Finally, to ensure that the choice of target protein did not unduly affect the results, we repeated the analysis 100 times, choosing a new target protein from the 1145 unique families containing experimentally annotated triples each time (685 with Biological Process and 711 families with Molecular Function annotation). Table 1 summarizes counts in the Biological Process and Molecular Function ontologies.

**Table 1:** Family-based analysis using functional similarity and sequence identity.

	*Biological Process*		*Molecular Function*	
	Paralog has higher functional similarity	Ortholog has higher functional similarity	Paralog has higher functional similarity	Ortholog has higher functional similarity
Paralog has higher sequence identity	17.4±0.2	3.6±0.1	17.7±0.2	7.2±0.1
Ortholog has higher sequence identity	442.4±0.8	221.6±0.8	346.8±0.9	339.3±0.9

Each field shows the average number of protein families (±standard error), out of 100 runs with randomly selected target proteins, in which the row and column conditions were satisfied.

The family-based analysis showed similar trends to those observed in previous sections. In the Biological Process category, if the orthologous sequence was more similar to the target protein, the ortholog had higher functional similarity to the target protein than all of its paralogs in only 33.4±0.1% of the cases (mean ± standard error). In contrast, in 82.9±0.4% of protein families in which a paralogous sequence was most similar to the target protein, it was also functionally most similar. In the Molecular Function category, the observed difference between orthologs and paralogs was similar: an ortholog had higher functional similarity to the target protein than all of its paralogs in only 49.5±0.1% of the cases. On the other hand, if the most similar sequence to a target protein was a paralog, the paralog was functionally most similar to the target protein in 71.1±0.5% of families.

### Intra- vs. interchromosomal duplications

**Figure 3:**
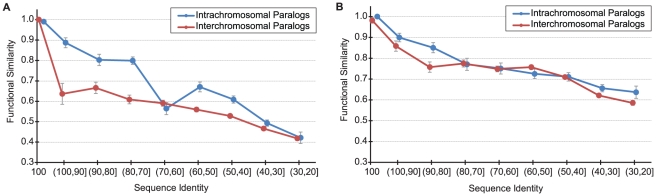
The relationship between functional similarity and sequence identity for paralogs on the same chromosome (blue) and on different chromosomes (red). Standard error bars are shown. (**A**) Biological Process ontology, (**B**) Molecular Function ontology.

It is known that paralogous sequences residing on the same chromosome are more likely to undergo non-allelic gene conversion in mammals [37], and are therefore more likely to maintain similar function due to concerted evolution. To explore this possibility, we examined the relationship between functional similarity and sequence identity for two types of gene duplication events: (i) those where the duplicated gene remains on the same chromosome (intrachromosomal), and (ii) those where the duplicated gene is moved to a different chromosome (interchromosomal). Figure 3 shows that a duplication event that places the new gene on a different chromosome reduces a protein's chances of retaining the original function. Interestingly, the extent of the functional divergence is statistically significant only in the Biological Process category, suggesting that while the biochemical function may be retained, the cellular context in which this function is utilized for a newly copied gene may be significantly different. Thus, gene transposition appears to be a viable evolutionary mechanism for mixing and matching protein molecular functions to attain more complex cellular functionalities.

### Case studies

We examined two families in further detail. (1) We compared the functional similarity of orthologs and paralogs in the full set of nuclear receptors in human and mouse, a well-studied group of proteins. Out of the 48 and 49 nuclear receptors identified in human and mouse, respectively [38], the Biological Process dataset contained 40 (20 human and 20 mouse) receptor proteins functionally annotated, and the Molecular Function dataset contained 46 (23 human and 23 mouse) functionally annotated proteins; these data include both orthologs and outparalogs, but unfortunately no inparalogs have been annotated. We counted the number of times either the ortholog or a paralog had higher functional similarity with each target protein in both the Biological Process and Molecular Function datasets (Table S1). In both datasets, a paralog was more functionally similar than the ortholog for the majority of the targets, and the specific paralog with the highest functional similarity was most often an outparalog in the same species (Table S1).

(2) Another example of a violation of the ortholog conjecture is found in the mitogen-activated protein kinase kinase kinase kinase 2 (MAP4K2) family. MAP4K2 is a serine/threonine protein kinase, expressed in lymph nodes, but also in other tissues such as lung, brain, and placenta [39]; its detailed function, however, remains incompletely understood. The MAP4K2 gene family consists of 1 mouse ortholog (*mMAP4K2*), 3 human outparalogs (*hMAP4K1, hMAP4K3, hMAP4K5*), and 3 mouse outparalogs (*mMAP4K1, mMAP4K3, mMAP4K5*) (Figure S3). Of these homologs, five have been experimentally annotated by functional terms in the Biological Process category. While the human *hMAP4K2* shares 94% sequence identity with its ortholog in mouse, their functional similarity is only 5% (45 annotated terms in human, 13 in mouse). In contrast, its functional similarity with its own outparalogs was 69% on average, including 82% similarity with *hMAP4K3*, a within-species outparalog.

### Addressing potential biases in the data

We analyzed multiple potential biases in the data that could impact the conclusions of this work. They included: 1) Functional annotation that is organism-specific, i.e. certain functions may be studied only in humans while others may be studied only in mice. To address this possibility we repeated our analysis using only the subset of functions studied in both human and mouse; there was no significant difference in the shape of the functional similarity curves relative to that shown in Figures 1 and 2 (Figure S4). 2) Different experimentalists may study protein functions at different levels of specificity according to the GO, resulting in functional annotations at very different levels of resolution. To address different specificities/depths of protein annotation, all functional terms deeper than the lowest maximal term depth over all proteins in a family were removed. That is, proteins annotated with more specific terms were generalized to the annotation depth of the protein that was annotated using the most general terms (the root node was excluded from the analysis). Again, the results of this analysis showed no significant differences in the shape of the functional similarity curves (Figure S5). Repeating our analysis using the generic GOslim ontology also did not affect the results (data not shown). 3) We hypothesized that proteins that were annotated in the same publication may have higher chances of being associated with the same functional terms, presumably due to unique inclinations of individual researchers. To get around this potential bias, we repeated our analysis while requiring that all proteins used in homologous pairs be annotated in a separate publication (based on the PubMed Identifier assigned to each functional annotation in the GO database). This analysis again showed no significant differences in trends (Figure S6). However, it did show that there are preferences toward the same annotation when multiple homologs were functionally annotated in the same article: functional similarity went up 0.1–0.3 across orthologs and paralogs for both Biological Process and Molecular Function. 4) We were concerned that different experimental methods would bias the set of annotation terms assigned to each gene. We therefore compared only those protein pairs that were annotated using the same GO evidence codes (Figure S7A). For Biological Process, there is very little difference from the complete dataset. For Molecular Function, there is a significant difference in the functional similarity of orthologs, increasing from an average of 0.65 to 0.85. However, we still observe higher levels of functional similarity for paralogs (Figure S7A). We also repeated our analyses without including the TAS (Traceable Author Statement) evidence code and found no qualitative difference in results (Figure S7B). 5) Finally, we speculated that it is possible that there is a reporting bias that may have influenced the functionally annotated orthologs, such that the genes present in the GO database are a non-representative subsample of all orthologous pairs between human and mouse. For instance, it is possible that experimental annotations for highly conserved orthologs are under-represented in the database because it is assumed that their functions are also highly conserved. However, the average sequence identity between 1-to-1 orthologs used in our analyses was similar to 1-to-1 orthologous pairs that were not functionally annotated and were therefore not included in our analysis (0.879 vs. 0.859 for Biological Process; 0.889 vs. 0.849 for Molecular Function). Thus, we believe that it is unlikely that a substantial reporting bias significantly influences the results of our analysis.

### Microarray-based measures of functional similarity

Because all of the above analyses are based on user-reported or curator-based determinations of function, they may still be affected by individual researcher biases that we cannot control for. The only way to avoid this potential problem is to obtain a measure of function that is not dependent on an individual's interpretation of experiments. Therefore, we conducted a parallel analysis of the relationship between protein similarity and functional similarity using microarray data from 25 homologous tissues in human and mouse [40].

**Figure 4:**
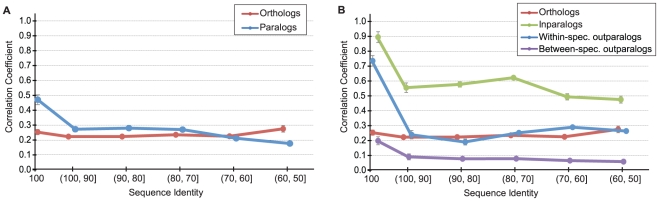
The relationship between the correlation in gene expression across 25 tissues (as measured by microarray) and sequence identity for (A) human-mouse orthologs (red) and all paralogs (blue), and (B) human-mouse orthologs (red), inparalogs (green), within-species outparalogs (blue), between-species outparalogs (purple). Standard error bars are shown.

We used the correlation in levels of normalized gene expression across tissues as our measure of functional similarity (see Materials and Methods). Our final microarray dataset included 10,863 orthologs and 21,780 paralogous comparisons of all types, consisting of 2,014 inparalogs, 10,396 within-species outparalogs, and 9,370 between-species outparalogs. Figure 4 shows the relationship between functional similarity and protein sequence identity for all pairs of genes represented in the microarray dataset. Consistent with all of the results obtained from the GO experimental dataset, microarray-based functional similarity shows a generally higher similarity between paralogs than orthologs (≥70%; *P*<0.01; Wilcoxon test) and a strong positive correlation with the sequence identity of paralogs but not orthologs. Our results were not dependent on the distance measure used to quantify functional similarity (see Materials and Methods). In addition, we again find that within-species paralogs—whether inparalogs or outparalogs—show the strongest relationship between sequence similarity and functional similarity.

The microarray data used here have also been utilized in a number of previous evolutionary studies, though these studies largely focused only on paralogs [41], only on orthologs [42], or on comparisons between orthologs with and without lineage-specific paralogs [43]. While these previous studies did not present their analyses in exactly the same way as we have done, we stress that for both paralogs and orthologs our results are in strong quantitative and qualitative agreement with these studies. For both the relationship between protein similarity and functional similarity, and for the average correlation in expression patterns, our results are consistent with previous results; that is, nothing about the way we have conducted our analysis has biased us toward our finding. We have largely followed the proscriptions of these previous papers for normalizing the microarray data and in controlling for cross-hybridization—which all of these previous papers agree does not appear to be an issue in these data.

Because there is no interpretation or assignment of functional terms needed to obtain these results, we believe they strongly support all of our previous analyses. It should also be noted that very few of the above GO-based analyses used expression evidence: in particular, there were only a total of 310 annotations that used the IEP evidence code for either the Molecular Function or Biological Process categories. Therefore, these two datasets are largely non-overlapping and provide independent support for the results.

## Discussion

The accelerating pace of whole-genome sequencing coupled with the rapid—but relatively slower—pace of functional genomics projects has required commensurately fast methods for computational annotation of genes and proteins. Because functional studies are disproportionately concentrated in only a handful of model organisms, the working model for computational annotation has been transfer-by-similarity [44], a principle in which experimentally determined functional annotation from a characterized protein is assigned to an uncharacterized protein if their sequence similarity is greater than some pre-specified threshold (e.g. sequence identity, E-value). With some caveats involving local vs. global sequence alignments (especially for multi-domain proteins), the basic tenet of such function transfer is that proteins that are closely related (and therefore similar in sequence) tend to have similar functions. Several recent papers have discussed the details of such annotation transfer, attempting to find similarity thresholds necessary for accurate inference of enzymatic functions [45],[46]. More sophisticated prediction algorithms, exploiting not only sequence similarity but also the structure of functional ontologies, have also been proposed [47], [48]. The field of phylogenomics [10], [13] uses evolutionary relationships as a guide to function prediction from sequence, preferentially transferring annotations between orthologs over paralogs because they are believed to be more functionally similar (the ortholog conjecture). Our study is the first to address this assumption using experimental evidence from 12,204 unique papers as well as an independent microarray dataset.

### The evolution of gene function

Our results strongly suggest that the ortholog conjecture is not correct between human and mouse: given equivalent levels of protein divergence (or even slightly higher divergence), paralogous genes from the same species (either human or mouse) are better predictors of function than are orthologs from the other species. A similar result was previously obtained among yeast, fly, and worm when comparing conserved protein-protein interactions between homologs within the same species and homologs from different species (although this study did not distinguish among orthologs, inparalogs, and outparalogs [49]). We ensured that our analyses were not affected by a large number of possible biases. We considered biases due to the ontology terms used in human and mouse, the depth of annotation terms used, whether homologs were studied in the same or different publications, biases due to differences in experimental procedures, and even biases in the user-defined interpretation of function. We found several interesting biases in the data—notably, that functions of homologs of any kind reported in the same publication or using the same experimental technique were more likely to be similar than a random pair of homologs of equal protein divergence—but none of these biases affected the qualitative patterns found in our data.

In addition to a general lack of support for the ortholog conjecture, our analyses revealed several surprising patterns. One of the most surprising is the lack of any discernible relationship between protein similarity and functional similarity for orthologs, whether considering Biological Process or Molecular Function annotations (Figure 1). Average functional similarity for orthologs is between 60–70%, regardless of level of divergence. Even for orthologous proteins approaching 100% identity, there is still relatively little overlap in annotation. While this fact may at first seem surprising, it is important to consider how individual experiments are conducted. Almost never are single genes (or proteins) from both mouse and human isolated and then compared in the same *in vitro* assay. Instead, the vast majority of experiments included in our dataset are conducted *in vivo* (e.g. knockouts, genetic crosses), *in situ* (e.g. tissue-specific expression), or *in vitro* but with species-specific conditions and/or interactors (e.g. yeast two-hybrid). Function is therefore assessed in the context of individual organisms, not in a common laboratory setting.

The importance of cellular and organismal context in defining protein function may go a long way toward explaining many aspects of our results, including the lack of a relationship between functional and sequence similarity for orthologs, the presence of this relationship for paralogs, and the differences between different types of paralogs (in-/outparalogs). We propose that the key to understanding the rate at which protein function evolves is not how quickly the protein sequence itself evolves, but rather the rate at which its cellular context—including directly and indirectly interacting molecules—evolves. To further explain this hypothesis, note that all of the orthologous pairs studied here are the same age: that is, they all share a last common ancestor at the split between the human and mouse lineages, regardless of their level of sequence identity. Unlike orthologs, the paralogs studied here shared common ancestors at many different times in the past, with some paralogs having split only a few million years ago while others split >100 million years ago. We propose that this difference in divergence times is the key to understanding the difference in relationships between functional and sequence similarity. The orthologs all share the same age—and therefore the same average functional similarity—but the paralogous pairs are of many different ages—and therefore different functional similarities.

Why should proteins of the same age share the same level of functional similarity? While there is no direct role for “time” in evolution that is not tied to mutation, we suggest that what time represents here is the evolution of the cellular context: the sum of the evolutionary changes over all of the directly and indirectly interacting molecules. If this context evolves at a steady rate (i.e. the average amount of functional change among all of the interacting molecules remains relatively constant), then protein function will appear to evolve at a steady rate, a rate largely disconnected from the level of an individual protein's sequence divergence. Several pieces of evidence support this conjecture. First, our results above show that even orthologous proteins that are 100% identical have different functions. Since it is obvious that the proteins themselves have not changed, the change must be due to regulation or downstream effects of these molecules. For example, Liao and Zhang [50] found that >20% of genes that are essential for viability in humans are not essential in mouse. It is unlikely that changes to the proteins themselves have made them essential or not, but rather that their context in cellular and organismal networks has evolved [50]. Second, we find a weak relationship between synonymous sequence identity—a good measure of divergence time [51]—and functional similarity for paralogous pairs (Figure S8). This supports the idea that time is a key factor in the evolution of protein function. Finally, we again note that there is higher functional similarity among inparalogs and within-species outparalogs than there is for either orthologs or between-species outparalogs. Because both inparalogs and within-species outparalogs are present in the same organism, it is highly likely that they share a much more similar cellular context. And because this context is highly similar, the functions of these proteins are also likely to be more similar. Our conclusion is that the most important aspect of functional similarity is not sequence similarity, but rather contextual similarity. A straightforward experiment to test this proposal would involve collecting functional data for orthologous pairs of different ages to see whether there is the predicted relationship between sequence identity and functional similarity. We would expect to see the same pattern in any pair of orthologs considered, of any age (cf. [49]).

Some researchers may be concerned that the function being measured here is not independent of the organism, and is therefore not appropriate for testing the ortholog conjecture. Of course it is possible that if measured in a common *in vitro* environment orthologous proteins really would be more functionally similar than paralogous proteins—after all, studies of rates of protein sequence evolution suggest an increased rate of sequence change among paralogs [20]–[23]. However, this is not the manner in which the vast majority of functional data is collected, and would therefore be little solace in applying the ortholog conjecture to real data.

### Implications for protein function prediction

The results of our study suggest that neither sequence similarity nor identification of orthologous assignments alone can be considered an accurate predictor of protein function. We find that orthologous proteins between human and mouse share a constant level of functional similarity over a wide range of (global) sequence identities, while the functional similarity between paralogs is dependent on the type of paralogy, level of sequence identity, relative chromosomal location of duplicated genes, and organismal context. We find that sequence identity thresholds as a means of function transfer are generally applicable only to within-species paralogs. Moreover, these thresholds depend on the type of paralogy and a specific duplication event, with inparalogs typically having lower thresholds for similarly accurate functional transfer than outparalogs. On the other hand, in the absence of within-species paralogs, our data indicates that orthologs and between-species outparalogs are similarly accurate in predicting protein function. In general, however, such relationships cannot be deemed ideal for function transfer of GO terms, as the average accuracy of predictions using orthologs and between-species outparalogs were consistently lower than 0.70 (Figure 1). Though many computational methods use only orthologous genes for function prediction, for methods that can be tuned to exploit different types of evolutionary relationships (e.g. SIFTER; [18]) our results can be used to improve prediction accuracy.

Functional annotation of genes with unknown function is often carried out by researchers working on particular proteins. In these cases—far from being an automated process of ortholog identification and functional transfer—individual researchers may examine the function of many closely related homologs before making decisions about functional annotations, or even before designing experiments. If they are available, researchers may be using the functions of both orthologs and paralogs to guide their own functional annotations. When inparalogs are available and happen to have the highest sequence identity, these genes may actually be the ones having the largest influence on the functional annotations in common databases; such a process of individual functional inference would create a pattern much like the one we observe. While our analysis of microarray data is consistent with the high functional similarity of within-species paralogs and is free from individual researcher or curator bias, we cannot rule out the possibility that such bias exists in widely used databases. However, such biases are likely to only apply to organisms already being studied by a large community of researchers in molecular biology. Many new genomes are being sequenced solely for the evolutionary or environmental importance of a species, and are therefore unlikely to have much prior data on gene and protein function. In these cases, our results suggest that functional transfer need not be dependent on the identification of orthologous genes in a model organism.

There are 31,479 proteins in the Swiss-Prot database with experimentally characterized function and 40,951 proteins in the Gene Ontology database (data as of February 1, 2010). The functions of this relatively small group of proteins have been transferred to a much larger number of homologous proteins and propagated across biological databases, often with gross inaccuracies [52]. Inaccurate functional annotation via computational methods can influence a wide variety of biological conclusions: for instance, any analysis looking for enriched or over-represented GO terms. We suggest that such studies should be cautiously interpreted until the prediction of protein function reaches the sensitivity and specificity necessary for accurate functional inference.

Finally, it must be mentioned again that our study has only addressed protein functions in two organisms, human and mouse. A fuller picture of the accuracy of protein function prediction would include many pairs of species from across the tree of life (see [49] for similar results from comparisons among yeast, fly, and worm). However, our study includes *human and mouse*: if the main purpose of biomedical research into model organisms is to understand the function of genes and proteins in humans, then we might expect these studies to be predictive of function in humans. While our results certainly show that mouse proteins are predictive of the function of human proteins (Figure 1), they also strongly suggest that the best model organism is ourselves.

## Materials and Methods

### Comparative genomics data

Ensembl Compara (release 49, March 2008) gene trees were used to identify all homologous human-human, mouse-mouse, and human-mouse gene pairs. Though there are many methods and databases available for identifying homologous relationships, they provide qualitatively similar results [53]. *Ortholog assignments:* Ensembl homology descriptions “ortholog 1:1” and “apparent ortholog 1:1” were used to annotate orthologous pairs. The apparent orthologs were treated as 1-to-1 orthologs since this description can result from a situation where a gene duplication is actually followed by gene losses in both lineages, but more often occurs because of an incorrect tree topology and incorrect duplication node labeling [33]. *Paralog assignments*: all between-species paralogs were treated as outparalogs. To distinguish inparalogs from outparalogs among the within-species paralogs, we examined the branch on the tree where the gene duplication took place to determine if the duplication occurred subsequent to the human-mouse speciation event. While the Compara dataset does not include bootstrap values for each node in the gene tree, incorrect trees will only conflate orthologs with between-species outparalogs and inparalogs with within-species outparalogs (because species assignments will never be mistaken). Though we cannot control for each type of error, the fact that within-species gene pairs cannot be confused with between-species gene pairs (of any kind) means that our main results are robust to the exact tree topologies. In total, our dataset consisted of 26,467 gene trees containing 22,137 human and 22,039 mouse genes (Dataset S1).

### Protein function data

Biological Process and Molecular Function protein function information was retrieved from the Gene Ontology (GO) database. Only the curated GO term annotations were used in the analysis. These include all experimentally inferred annotations: inferred from direct assay (IDA), expression pattern (IEP), genetic interaction (IGI), mutant phenotype (IMP), and physical interaction (IPI) evidence codes. We also included the traceable author statement (TAS) and inferred by curator (IC) evidence codes. Since both the Biological Process and Molecular Function ontologies are represented by directed acyclic graphs (DAGs), the original functional terms were propagated towards the root of each DAG (with the root node excluded) thus producing a complete set of terms for each protein. The GO seqdblite database (release 2009-01-18) was used for term propagation. In total, 4,854 human and 4,089 mouse proteins had functional annotation in at least one GO DAG. This reduced the number of gene trees with at least two functionally annotated genes to 2,448; the total number of ortholog pairs is 2,579, inparalog pairs is 597, within-species outparalogs is 11,334, and between-species outparalogs is 9,840 (Dataset S1).

### Microarray data

Microarray data presented in Su et al. [40] was retrieved from the Gene Expression Omnibus, accession GSE1133. The data were collected on three different microarray platforms, two from human and one from mouse. The two platforms from human, GPL96 and GPL1074, consist of expression values in 78 tissues for 22,283 and 11,391 probesets respectively. The mouse platform, GPL1073, consists of expression values in 61 tissues for 31,373 probesets. 25 of these tissues are common between human and mouse and were used here. In order to create an updated mapping between probesets and genes, individual probe sequences (there are 16 per probeset) were searched against Ensembl transcripts using exact matches returned from BLAST. Only probesets that perfectly matched a gene's sequence and did not have probes matching any other gene were considered. When multiple probesets uniquely matched to the same gene, the values were averaged after normalization to give a single genic expression value.

Expression data was normalized within each platform individually. Expression values were first normalized within each individual tissue using the *z*-score method, forcing expression values within a tissue to have a mean of 0 and a standard deviation of 1. After expression values were normalized within a tissue, they were again normalized for individual probesets across tissues, forcing expression values for a single probeset to have a mean of 0 and a standard deviation of 1 across tissues. Specifically, if we represent the expression value of a probeset *i* in a tissue *j* as *s_ij_*, we can define the tissue-normalized expression value, *t_ij_*, as:
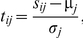
where *μ_j_* and *σ_j_* are the mean and standard deviation of expression values in tissue *j*. The final normalized expression value for a probeset *i* in tissue *j*, *n_ij_*, is defined as:
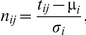
where *μ_i_* and *σ_i_* are the mean and standard deviation of *t_i·_* values for gene *i* in all tissues. After these two steps of normalization, we averaged probesets that match to the same gene and then averaged duplicate samples for the same tissue.

In total, we were able to obtain expression data for 15,907 human genes and 15,552 mouse genes. This reduced the number of gene trees with at least two functionally annotated genes to 7,495; the total number of data pairs used for orthologs is 10,863, for inparalog pairs is 2,014, for within-species outparalogs is 10,396, and for between-species outparalogs is 9,370 (Dataset S2).

### Calculation of similarity

We calculated protein sequence identity by using Needleman-Wunsch alignments of protein sequences with the Blosum62 scoring matrix (gap opening penalty  =  11; gap extension penalty  =  1). We divided the number of matching residues by the length of the alignment. For the calculation of *d*_N_/*d*_S_ and *d*_S_, we used the Goldman and Yang method [54].

To calculate functional similarity for the GO data, let *T*(*p*) be a set of propagated GO terms for protein *p* and *T*(*q*) be a set of propagated GO terms for protein *q*. Then, the functional similarity *fs*(*p*, *q*) between *p* and *q* was calculated as:
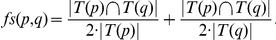



This formula can be interpreted as the average of the fraction of correctly predicted functional terms in *p* when protein *q* is used to predict *p*'s function (by transfer of all its terms), and the fraction of correctly predicted functional terms in *q* when protein *p* is used to predict *q*'s function [55]. This measure of functional similarity is known as the Maryland bridge coefficient and is highly correlated with the Jaccard coefficient—the size of the intersection over the size of the union between two sets [56]. Clearly, 0≤*fs*(*p*,*q*)≤1, with 0 corresponding to proteins with disjoint sets of functional terms and 1 corresponding to proteins with identical sets of terms. Functional similarity of 0 may occur because we removed the root node from each ontology.

To calculate functional similarity for the microarray data, we used the Pearson correlation coefficient (the Euclidean distance provided similar results). The correlation coefficient *corr*(*p*, *q*) between genes *p* and *q* (in a somewhat abused notation where *p* and *q* represent both genes and their indices in microarrays) for normalized data was calculated as:
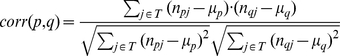
where *T* is the index set of tissues being considered, *n_pj_* is the normalized expression for gene *p* in tissue *j*, and *µ_p_* is the mean expression level for gene *p* over all tissues in *T*.

## Supporting Information

Figure S1 Different types of homology relationships among genes. **A)** The figure shows four hypothetical genes in humans (H1–H4) and two in mouse (M1–M2). There are four types of homologs shown: 1) M1 is an ortholog of H1, H2, and H3 because their last common ancestor is a speciation event (one-to-many orthology). 2) H1 is an inparalog of H2 and H3, with respect to the human-mouse split, because their last common ancestor is a duplication event more recent than the human-mouse split. 3) M1 is a within-species outparalog of M2 because they are related by a duplication event that occurred before the human-mouse split. 4) M1 is also a between-species outparalog of H4 because they are related by a duplication event before the human-mouse split (and in different genomes). **B)** The figure shows two hypothetical genes in humans (H1 and H2) and two in mouse (M1 and M2). There are three types of homologs shown: 1) M1 and H1 are one-to-one orthologs, as are M2 and H2. 2) M1 is a within-species outparalog of M2 because they are related by a duplication event that occurred before the human-mouse split, as are H1 and H2. 3) M1 is a between-species outparalog of H2 because they are related by a duplication event before the human-mouse split (and in different genomes), as are H1 and M2.
(PDF)

Figure S2 The relationship between functional similarity and *d*_N_ */d*_S_ calculated using the Goldman and Yang method. **A**) human-mouse orthologs (red) and all paralogs (blue). **B**) human-mouse orthologs (red), inparalogs (green), within-species (W-s) outparalogs (blue), between-species (B-s) outparalogs (purple). Counts of gene pairs in each bin are listed below each figure. Note that estimates of *d*_S_ (and therefore *d*_N_ */d*_S_) are inaccurate for long divergence times due to multiple substitutions at the same site; this likely affects the values for outparalogs.
(PDF)

Figure S3 The phylogenetic relationships between functionally annotated members of the MAP4K family, and counts of overlapping and non-overlapping GO terms for the target protein human MAP4K2 (red circles) and each of its homologs (blue circles). Tree branch lengths are not drawn to scale.
(PDF)

Figure S4 The relationship between functional similarity and sequence identity using only the subset of GO terms assigned to at least one human and at least one mouse protein. **A**) human-mouse orthologs (red) and all paralogs (blue). **B**) human-mouse orthologs (red), inparalogs (green), within-species (W-s) outparalogs (blue), between-species (B-s) outparalogs (purple). Counts of gene pairs in each bin are listed below each figure.
(PDF)

Figure S5 The relationship between functional similarity and sequence identity using a constant GO term annotation depth for all members of the gene family. For each family, the maximum depth of annotation (measured as the distance from the root node) for each protein was calculated, and then the minimum of the individual maximum annotation depths was found. All GO terms below this minimum were removed for all proteins in the family. **A**) human-mouse orthologs (red) and all paralogs (blue). **B**) human-mouse orthologs (red), inparalogs (green), within-species (W-s) outparalogs (blue), between-species (B-s) outparalogs (purple). Counts of gene pairs in each bin are listed below each figure.
(PDF)

Figure S6 The relationship between functional similarity and sequence identity excluding all GO term annotations derived from the same publication (based on PubMed ID) for both members of the homologous protein pair. During annotation, the same GO term can be assigned to a protein by two or more distinct PubMed IDs. In these cases, GO term annotations were not considered to have come from the same publication if different PubMed IDs could be assigned to the annotations for each member of the pair. **A**) human-mouse orthologs (red) and all paralogs (blue). **B**) human-mouse orthologs (red), inparalogs (green), within-species (W-s) outparalogs (blue), between-species (B-s) outparalogs (purple). Parts **C**) and **D**) show the same relationship using only GO term annotations derived from the same publication (based on PubMed ID) for both members of the homologous protein pair. Counts of gene pairs in each bin are listed below each figure.
(PDF)

Figure S7 The relationship between functional similarity and sequence identity using only protein pairs annotated with GO terms assigned by the same evidence code. All experimental (IDA, IEP, IGI, IMP, IPI), curator inferred (IC), and traceable author statement (TAS) evidence codes are included. **A**) human-mouse orthologs (red) and all paralogs (blue). **B**) human-mouse orthologs (red), inparalogs (green), within-species (W-s) outparalogs (blue), between-species (B-s) outparalogs (purple). Parts **C**) and **D**) show the same relationship using all experimental and curator inferred evidence codes, but excluding traceable author statements (TAS). Counts of gene pairs in each bin are listed below each figure.
(PDF)

Figure S8 The relationship between functional similarity and *d*_S_ calculated using the Goldman and Yang method for inparalogs only.
(PDF)

Table S1 Functional similarity within the nuclear receptor family in human and mouse. Of the total number of annotated proteins with both an ortholog and a paralog, the counts show the number in each category. Paralogs with higher functional similarity are further distinguished by whether the within-species or between-species outparalog was most similar.
(DOC)

Dataset S1 Measures of functional similarity, sequence similarity, and homology relationships between proteins, as well as GO codes associated with each protein used in the study.
(TXT)

Dataset S2 Correlation in gene expression profiles between proteins, tissues used from the human and mouse array experiments, mappings of probesets to genes, as well as normalized expression values for each gene.
(TXT)
