# Supplementary figures and images for: Testing the Ortholog Conjecture with Comparative Functional Genomic Data from Mammals
Source: PLoS Comput Biol. 2011 Jun 9;7(6):e1002073. doi: 10.1371/journal.pcbi.1002073 (PMC3111532; doi:10.1371/journal.pcbi.1002073)

Figure S1A

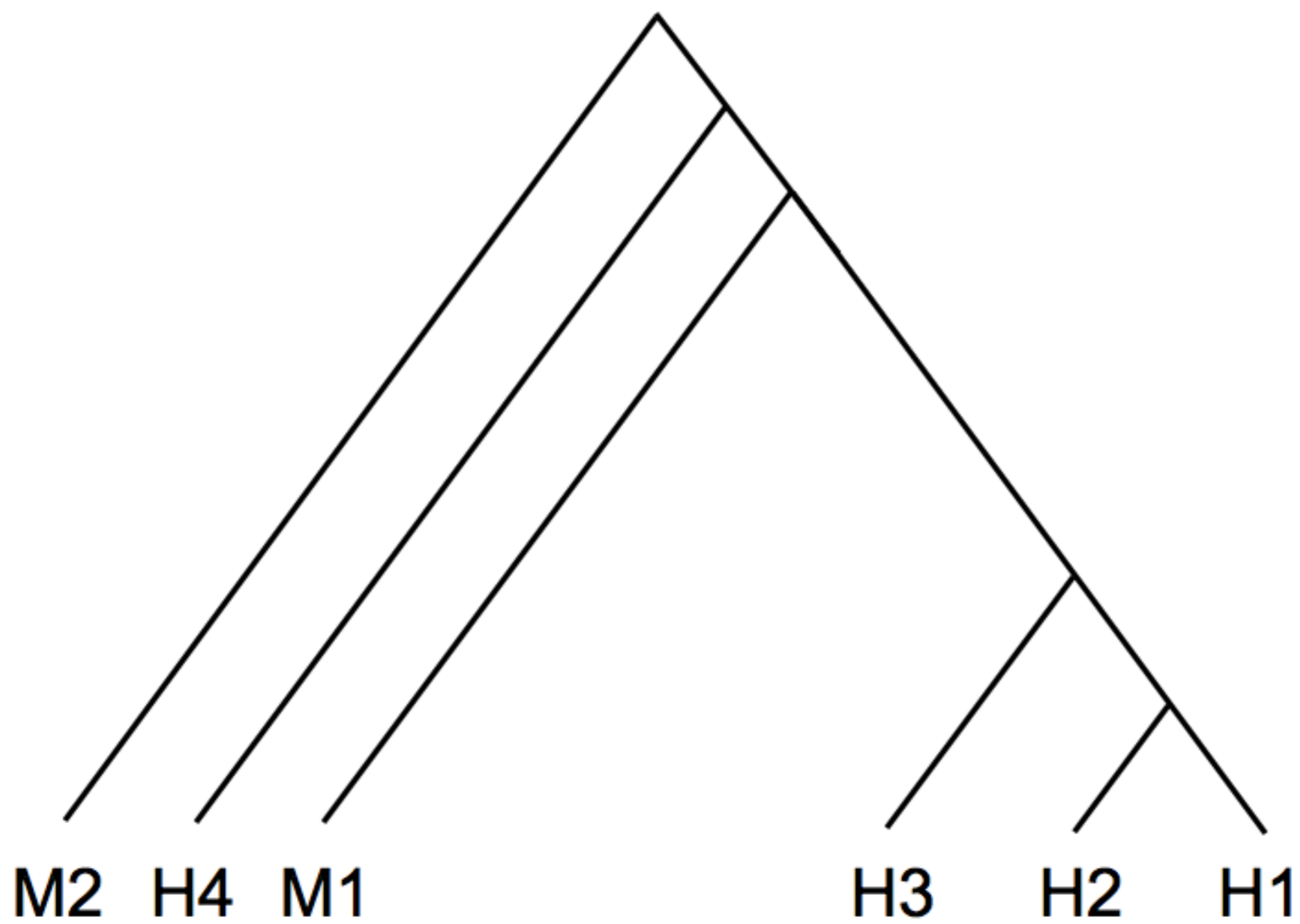

Figure S1B

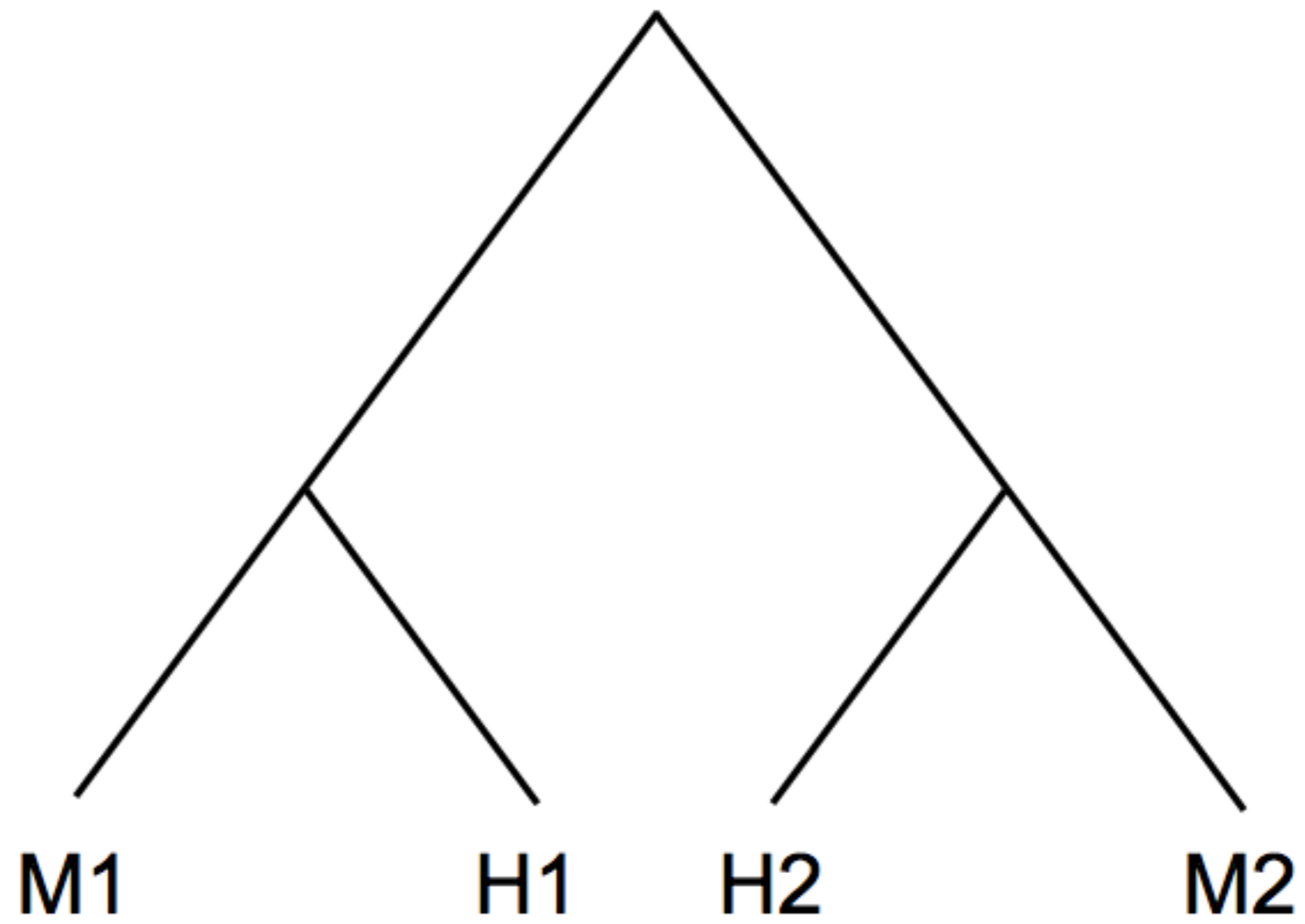

Supplement: Figure S1 — Different types of homology relationships among genes. A) The figure shows four hypothetical genes in humans (H1–H4) and two in mouse (M1–M2). There are four types of homologs shown: 1) M1 is an ortholog of H1, H2, and H3 because their last common ancestor is a speciation event (one-to-many orthology). 2) H1 is an inparalog of H2 and H3, with respect to the human-mouse split, because their last common ancestor is a duplication event more recent than the human-mouse split. 3) M1 is a within-species outparalog of M2 because they are related by a duplication event that occurred before the human-mouse split. 4) M1 is also a between-species outparalog of H4 because they are related by a duplication event before the human-mouse split (and in different genomes). B) The figure shows two hypothetical genes in humans (H1 and H2) and two in mouse (M1 and M2). There are three types of homologs shown: 1) M1 and H1 are one-to-one orthologs, as are M2 and H2. 2) M1 is a within-species outparalog of M2 because they are related by a duplication event that occurred before the human-mouse split, as are H1 and H2. 3) M1 is a between-species outparalog of H2 because they are related by a duplication event before the human-mouse split (and in different genomes), as are H1 and M2. (PDF) [file pcbi.1002073.s001.pdf]

Figure S3

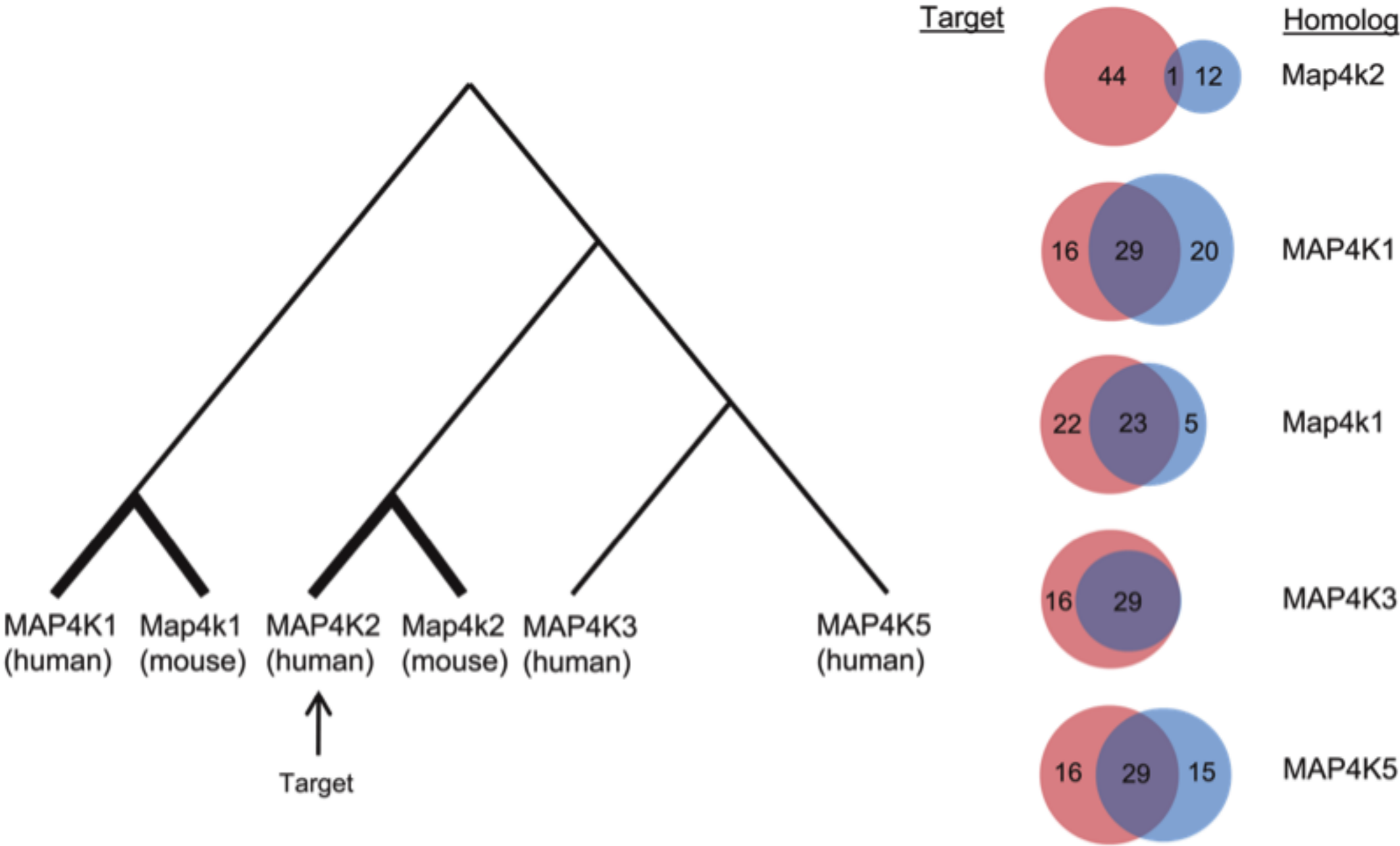

Supplement: Figure S3 — The phylogenetic relationships between functionally annotated members of the MAP4K family, and counts of overlapping and non-overlapping GO terms for the target protein human MAP4K2 (red circles) and each of its homologs (blue circles). Tree branch lengths are not drawn to scale. (PDF) [file pcbi.1002073.s003.pdf]

Figure S8

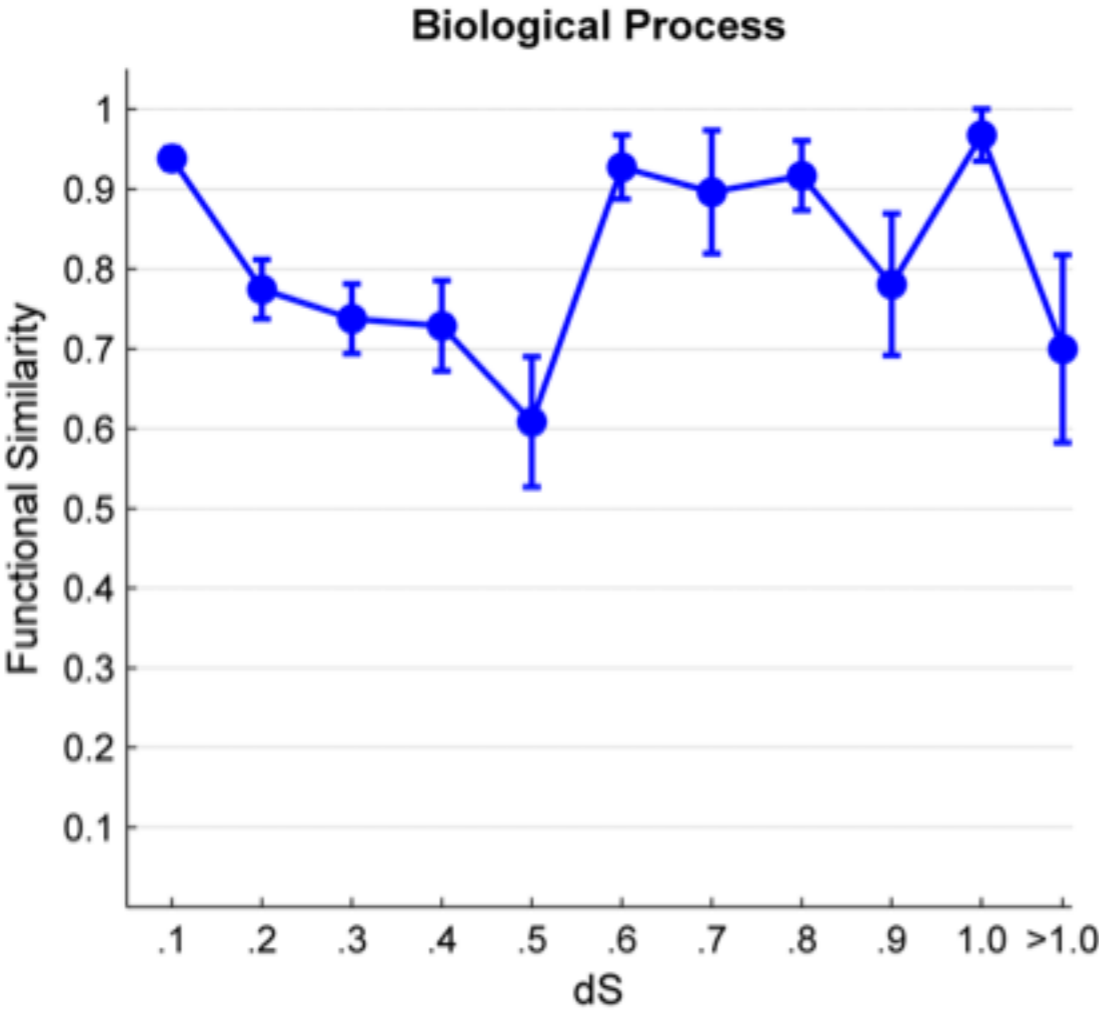

Inpara: (449) Bins: (162)(76)(50)(41)(25)(30)(13)(10)(12)(19)(11)

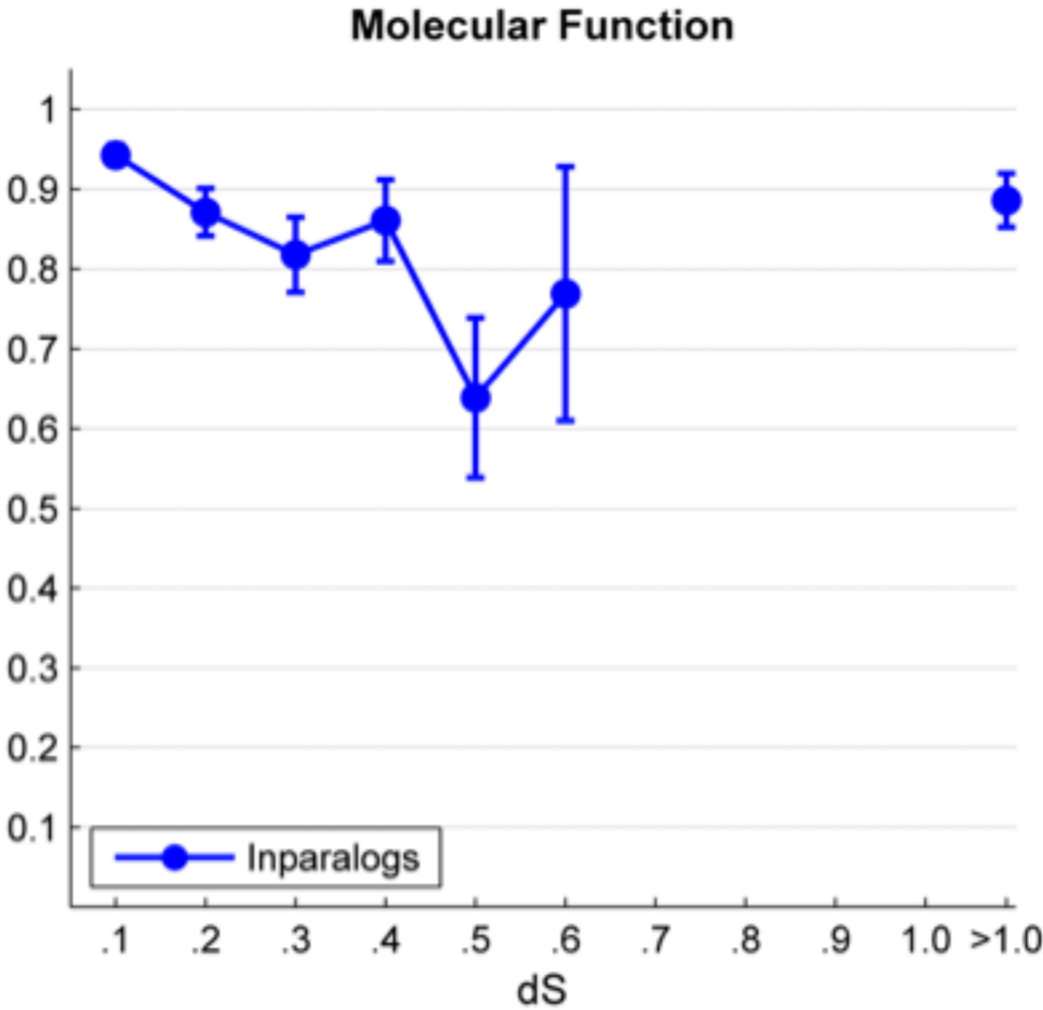

Inpara: (308) Bins: (156)(54)(36)(25)(14)(6)(0)(0)(0)(0)(17)

Supplement: Figure S8 — The relationship between functional similarity and d S calculated using the Goldman and Yang method for inparalogs only. (PDF) [file pcbi.1002073.s008.pdf]
