# Supplementary material for: Testing the Ortholog Conjecture with Comparative Functional Genomic Data from Mammals
Source: PLoS Comput Biol. 2011 Jun 9;7(6):e1002073. doi: 10.1371/journal.pcbi.1002073 (PMC3111532; doi:10.1371/journal.pcbi.1002073)
Supplement: Figure S2 — The relationship between functional similarity and d N /d S calculated using the Goldman and Yang method. A) human-mouse orthologs (red) and all paralogs (blue). B) human-mouse orthologs (red), inparalogs (green), within-species (W-s) outparalogs (blue), between-species (B-s) outparalogs (purple). Counts of gene pairs in each bin are listed below each figure. Note that estimates of d S (and therefore d N /d S) are inaccurate for long divergence times due to multiple substitutions at the same site; this likely affects the values for outparalogs. (PDF) [file pcbi.1002073.s002.pdf]

Figure S2A

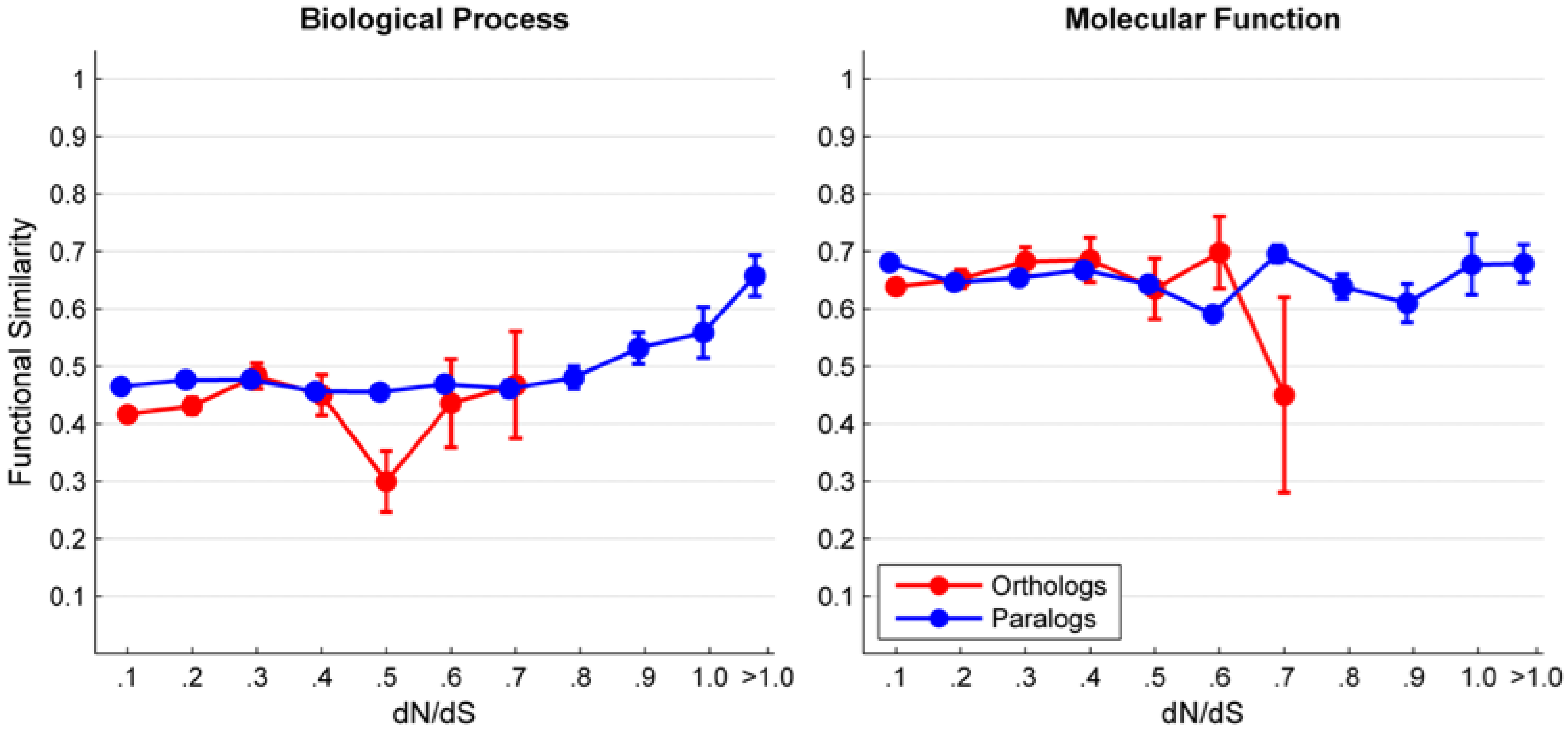

Ortho: (1804) Bins: (1075)(431)(167)(83)(27)(13)(8)(0)(0)(0)(0)
Para: (11225) Bins: (3372)(1419)(1658)(1563)(1309)(832)(514)(269)(138)(57)(94)

Ortho: (1767) Bins: (1096)(424)(137)(65)(27)(12)(6)(0)(0)(0)(0)
Para: (10613) Bins: (3426)(1421)(1523)(1458)(1140)(731)(454)(231)(106)(44)(79)

Figure S2B

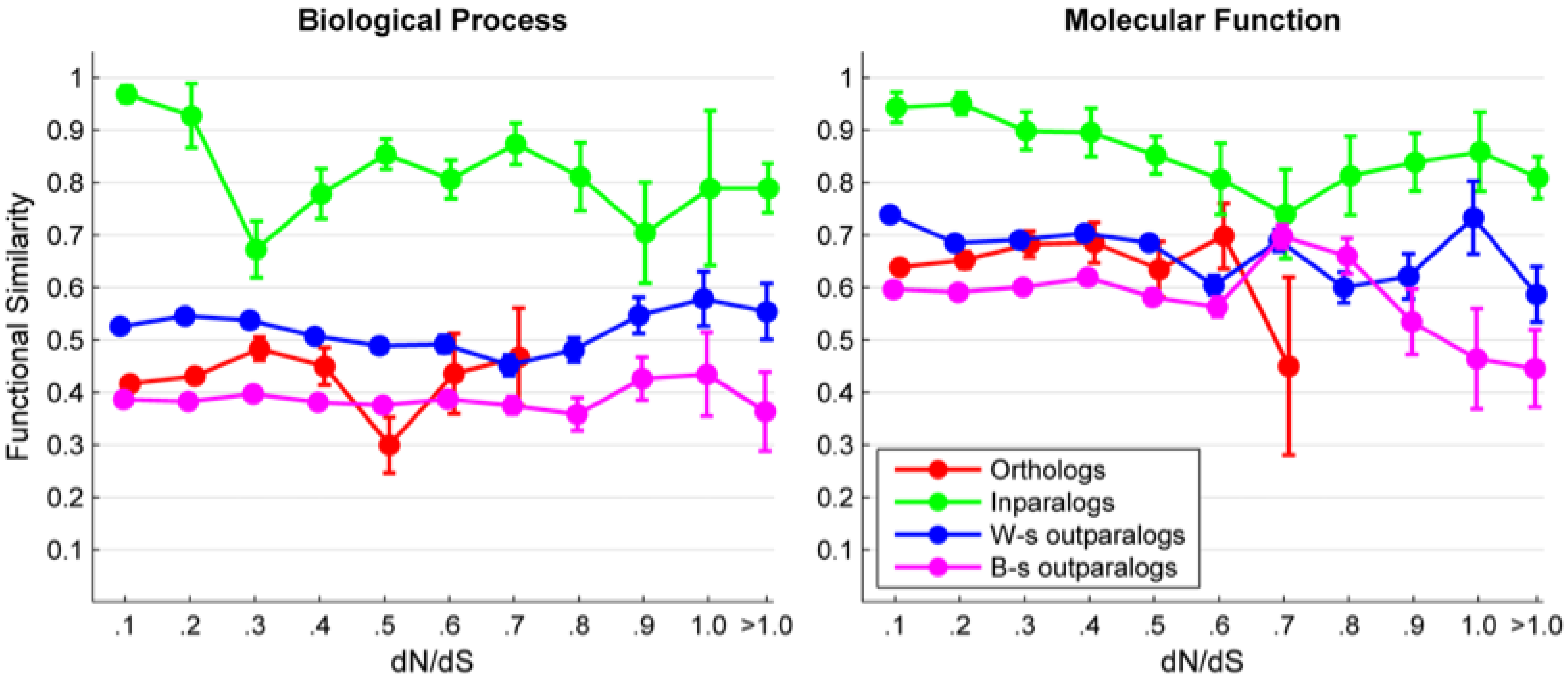

Ortho: (1804) Bins: (1075)(431)(167)(83)(27)(13)(8)(0)(0)(0)(0)
Inpara: (395) Bins: (27)(9)(27)(38)(66)(67)(51)(31)(21)(7)(51)
W-s outpara: (5817) Bins: (1784)(784)(887)(811)(637)(383)(243)(153)(72)(32)(31)
B-s outpara: (5013) Bins: (1561)(626)(744)(714)(606)(382)(220)(85)(45)(18)(12)

Ortho: (1767) Bins: (1096)(424)(137)(65)(27)(12)(6)(0)(0)(0)(0)
Inpara: (257) Bins: (39)(15)(29)(24)(38)(20)(21)(18)(8)(6)(39)
W-s outpara: (5681) Bins: (1922)(779)(801)(763)(574)(373)(220)(129)(64)(26)(30)
B-s outpara: (4675) Bins: (1465)(627)(693)(671)(528)(338)(213)(84)(34)(12)(10)
