# Supplementary material for: Testing the Ortholog Conjecture with Comparative Functional Genomic Data from Mammals
Source: PLoS Comput Biol. 2011 Jun 9;7(6):e1002073. doi: 10.1371/journal.pcbi.1002073 (PMC3111532; doi:10.1371/journal.pcbi.1002073)
Supplement: Figure S4 — The relationship between functional similarity and sequence identity using only the subset of GO terms assigned to at least one human and at least one mouse protein. A) human-mouse orthologs (red) and all paralogs (blue). B) human-mouse orthologs (red), inparalogs (green), within-species (W-s) outparalogs (blue), between-species (B-s) outparalogs (purple). Counts of gene pairs in each bin are listed below each figure. (PDF) [file pcbi.1002073.s004.pdf]

Figure S4A

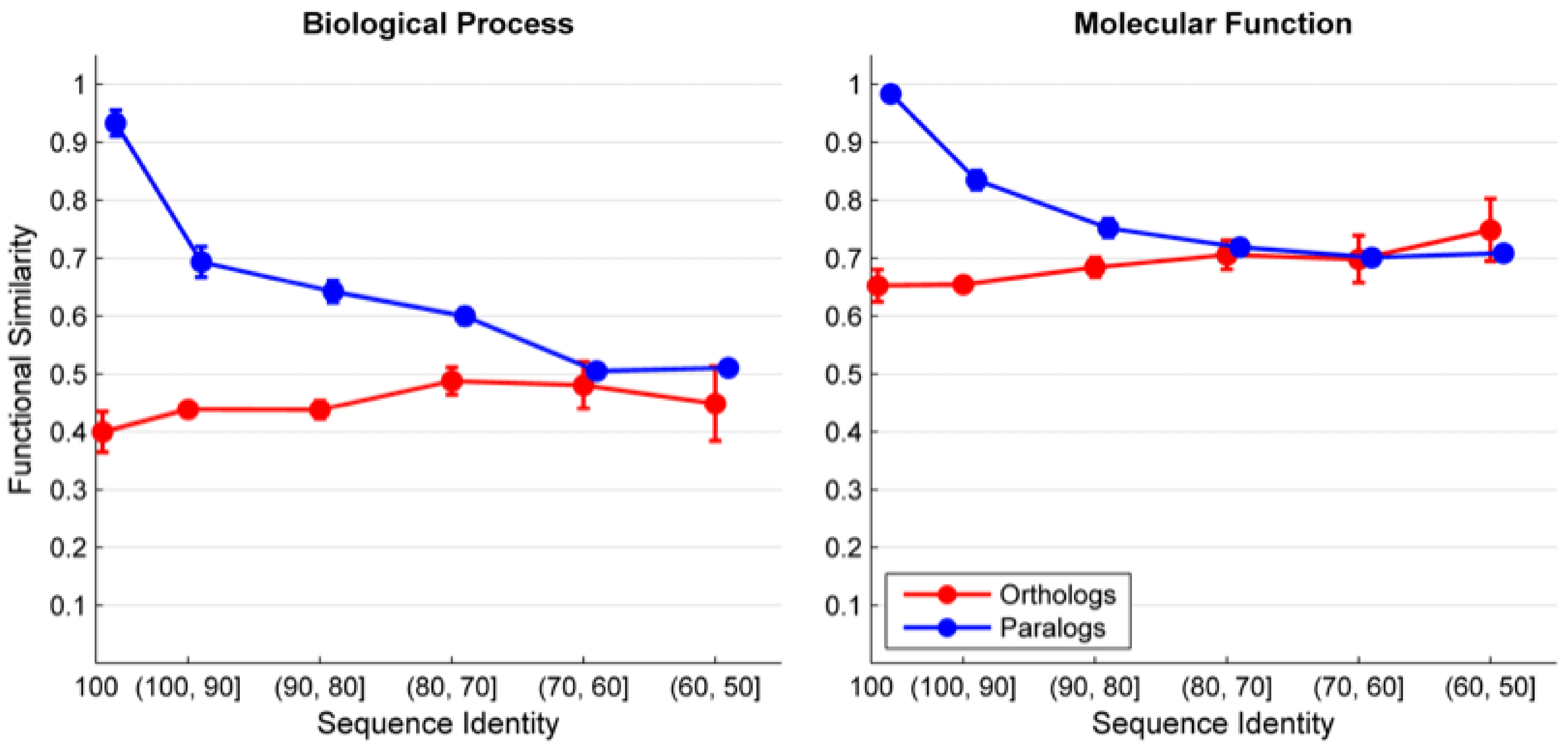

Orthologs: (1563) Bins: (73) (812) (411) (167) (77) (23)
Paralogs: (3805) Bins: (94) (173) (322) (686) (925) (1605)

Orthologs: (1601) Bins: (104) (870) (387) (152) (63) (25)
Paralogs: (4097) Bins: (281) (241) (385) (603) (1002) (1585)

Figure S4B

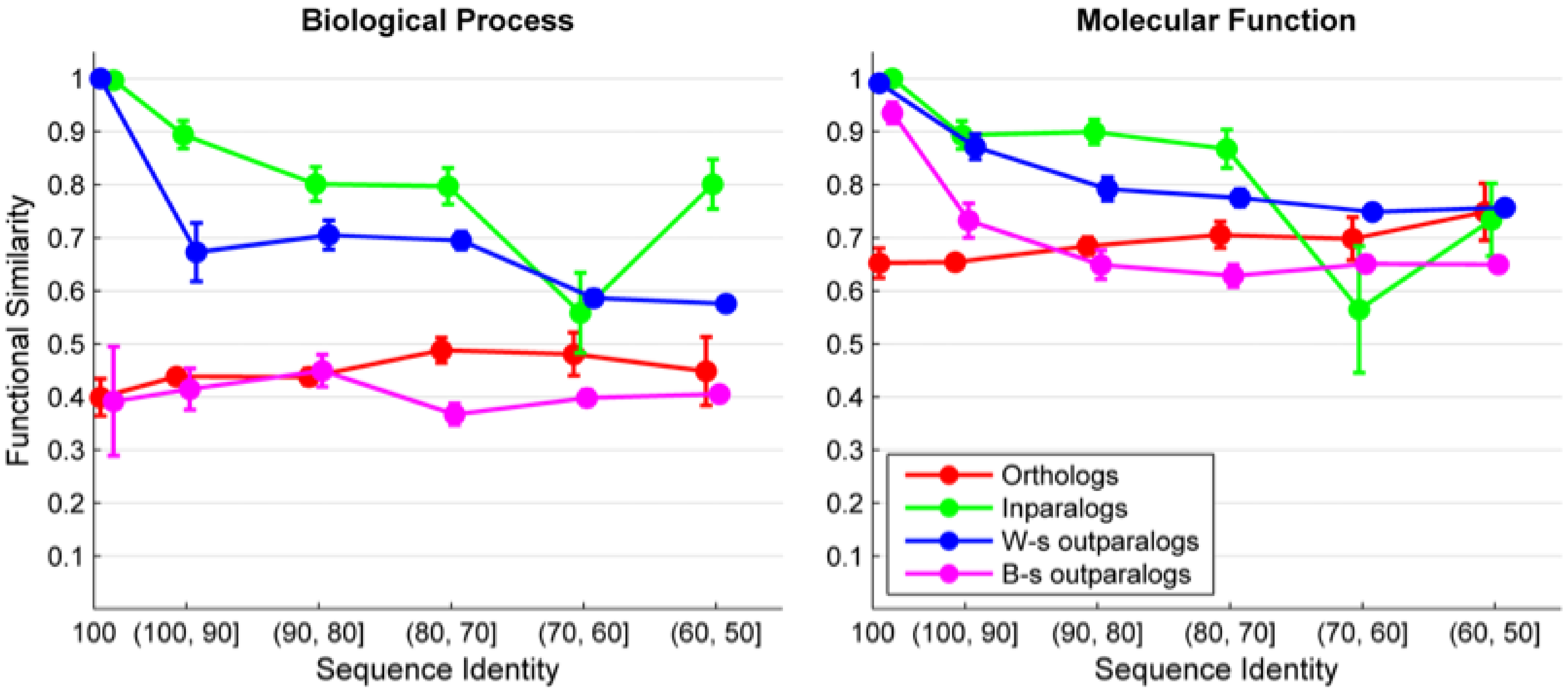

Orthologs: (1563) Bins: (73) (812) (411) (167) (77) (23)
Inparalogs: (400) Bins: (75) (78) (79) (82) (25) (61)
W-s outparalogs: (1915) Bins: (9) (42) (134) (379) (503) (848)
B-s outparalogs: (1490) Bins: (10) (53) (109) (225) (397) (696)

Orthologs: (1601) Bins: (104) (870) (387) (152) (63) (25)
Inparalogs: (268) Bins: (77) (62) (65) (35) (15) (14)
W-s outparalogs: (2123) Bins: (151) (105) (163) (315) (530) (859)
B-s outparalogs: (1706) Bins: (53) (74) (157) (253) (457) (712)
