# Supplementary material for: Testing the Ortholog Conjecture with Comparative Functional Genomic Data from Mammals
Source: PLoS Comput Biol. 2011 Jun 9;7(6):e1002073. doi: 10.1371/journal.pcbi.1002073 (PMC3111532; doi:10.1371/journal.pcbi.1002073)
Supplement: Figure S5 — The relationship between functional similarity and sequence identity using a constant GO term annotation depth for all members of the gene family. For each family, the maximum depth of annotation (measured as the distance from the root node) for each protein was calculated, and then the minimum of the individual maximum annotation depths was found. All GO terms below this minimum were removed for all proteins in the family. A) human-mouse orthologs (red) and all paralogs (blue). B) human-mouse orthologs (red), inparalogs (green), within-species (W-s) outparalogs (blue), between-species (B-s) outparalogs (purple). Counts of gene pairs in each bin are listed below each figure. (PDF) [file pcbi.1002073.s005.pdf]

Figure S5A

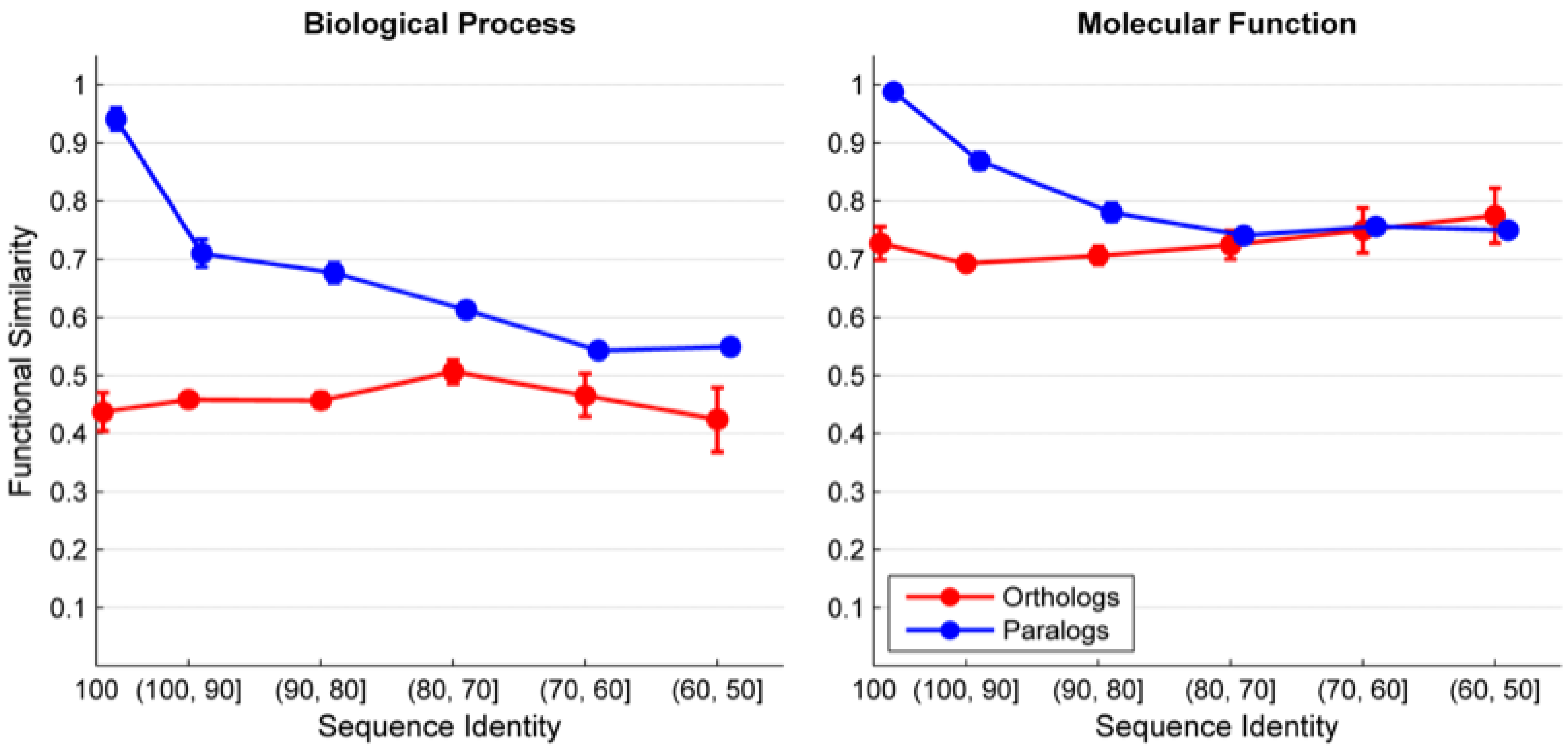

Orthologs: (1796) Bins: (79) (919) (476) (203) (89) (30)
Paralogs: (4202) Bins: (98) (184) (342) (755) (1016) (1807)

Orthologs: (1761) Bins: (105) (949) (433) (176) (70) (28)
Paralogs: (4568) Bins: (295) (261) (414) (683) (1120) (1795)

Figure S5B

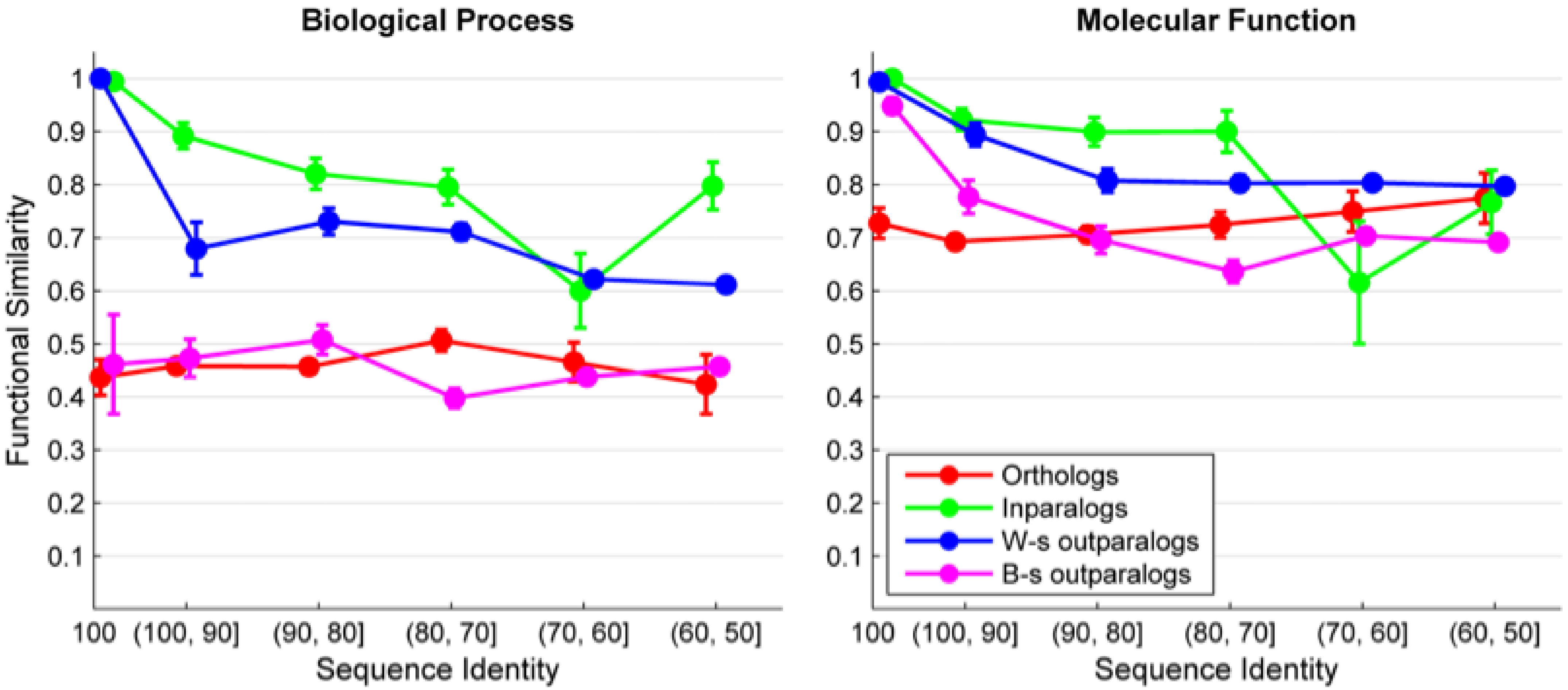

Orthologs: (1796) Bins: (79) (919) (476) (203) (89) (30)
Inparalogs: (418) Bins: (79) (82) (81) (87) (25) (64)
W-s outparalogs: (2103) Bins: (9) (45) (145) (407) (558) (939)
B-s outparalogs: (1681) Bins: (10) (57) (116) (261) (433) (804)

Orthologs: (1761) Bins: (105) (949) (433) (176) (70) (28)
Inparalogs: (313) Bins: (91) (76) (76) (39) (16) (15)
W-s outparalogs: (2383) Bins: (151) (109) (174) (367) (604) (978)
B-s outparalogs: (1872) Bins: (53) (76) (164) (277) (500) (802)
