# Supplementary material for: Testing the Ortholog Conjecture with Comparative Functional Genomic Data from Mammals
Source: PLoS Comput Biol. 2011 Jun 9;7(6):e1002073. doi: 10.1371/journal.pcbi.1002073 (PMC3111532; doi:10.1371/journal.pcbi.1002073)
Supplement: Figure S6 — The relationship between functional similarity and sequence identity excluding all GO term annotations derived from the same publication (based on PubMed ID) for both members of the homologous protein pair. During annotation, the same GO term can be assigned to a protein by two or more distinct PubMed IDs. In these cases, GO term annotations were not considered to have come from the same publication if different PubMed IDs could be assigned to the annotations for each member of the pair. A) human-mouse orthologs (red) and all paralogs (blue). B) human-mouse orthologs (red), inparalogs (green), within-species (W-s) outparalogs (blue), between-species (B-s) outparalogs (purple). Parts C) and D) show the same relationship using only GO term annotations derived from the same publication (based on PubMed ID) for both members of the homologous protein pair. Counts of gene pairs in each bin are listed below each figure. (PDF) [file pcbi.1002073.s006.pdf]

Figure S6A

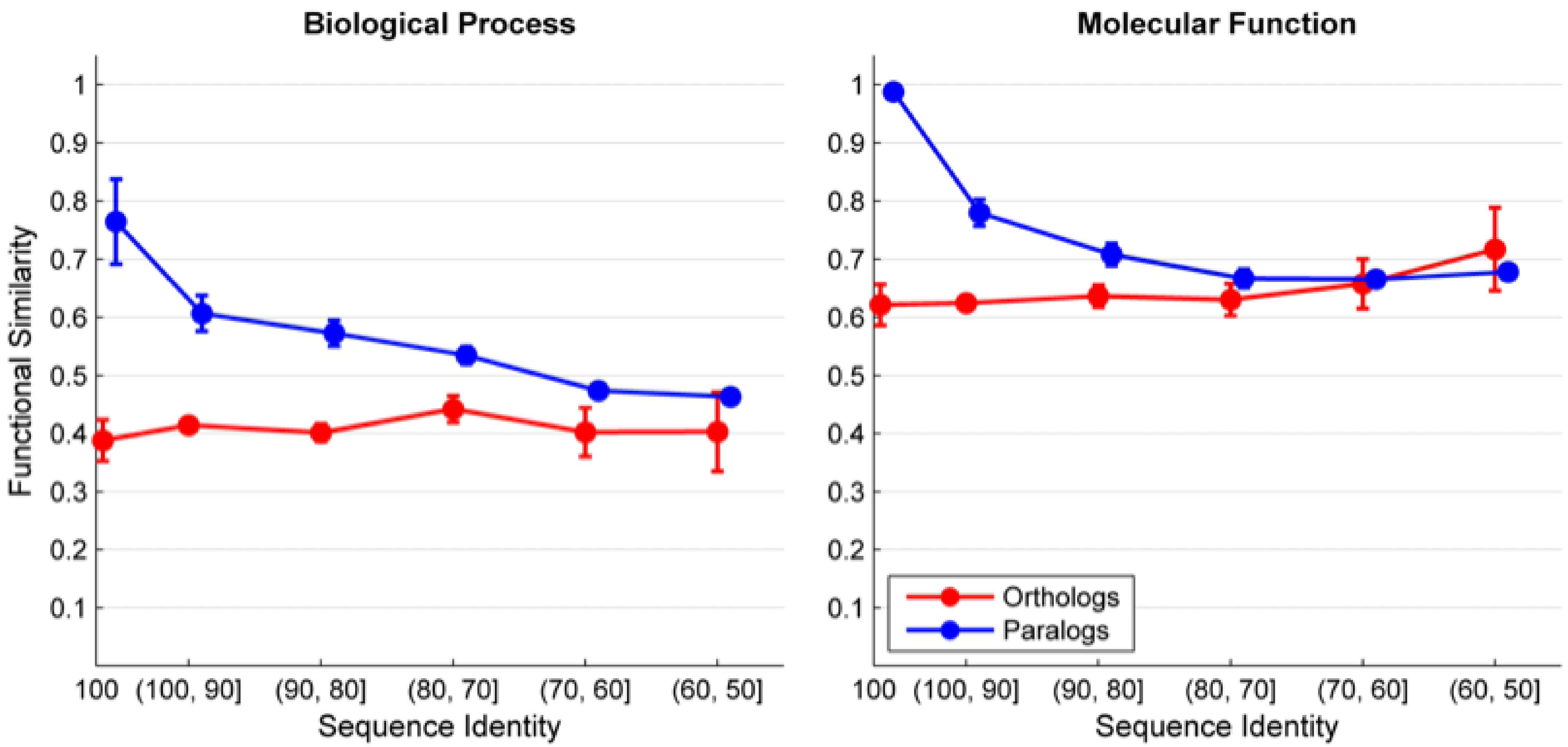

Orthologs: (1403) Bins: (65) (736) (373) (145) (62) (22)
Paralogs: (3059) Bins: (24) (119) (224) (543) (755) (1394)

Orthologs: (1316) Bins: (72) (725) (316) (132) (54) (17)
Paralogs: (3183) Bins: (143) (161) (300) (448) (817) (1314)

Figure S6B

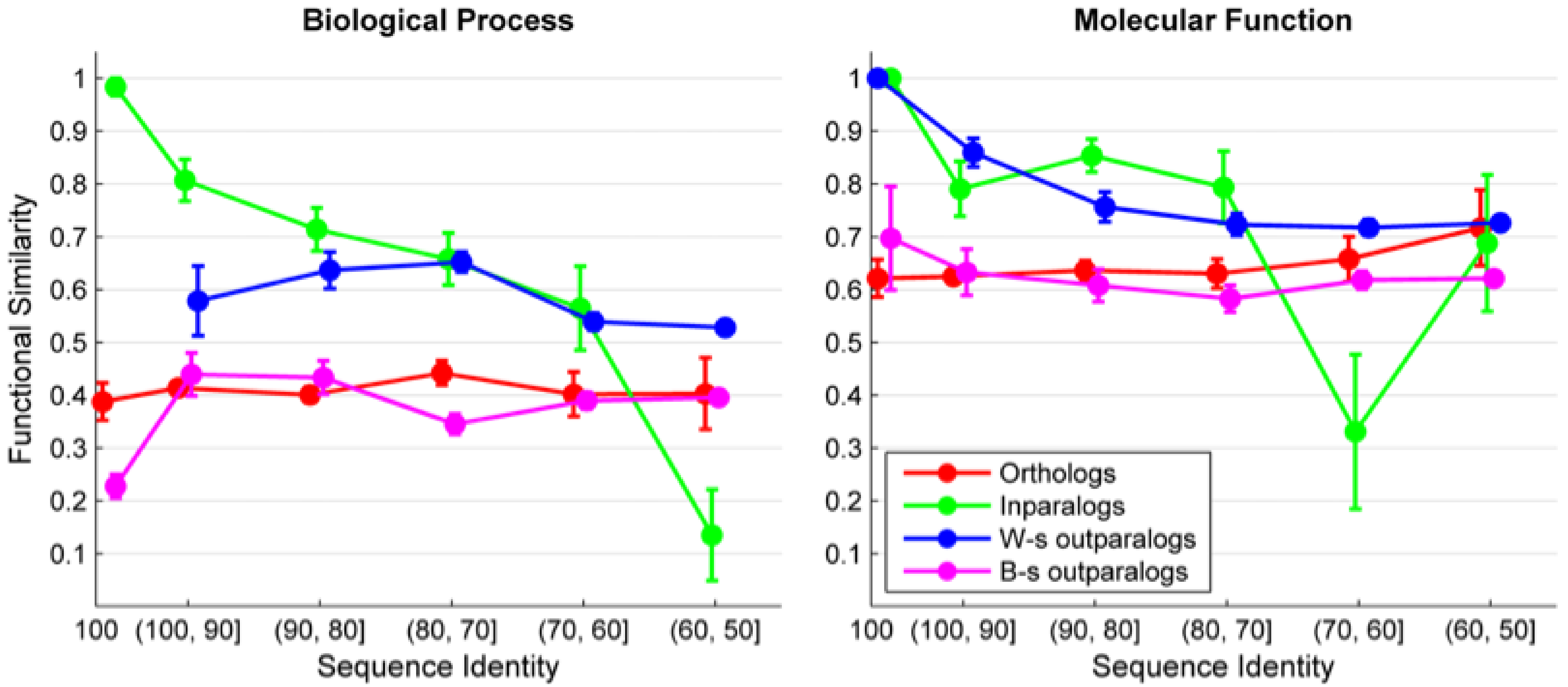

Orthologs: (1403) Bins: (65) (736) (373) (145) (62) (22)
Inparalogs: (157) Bins: (15) (42) (43) (28) (19) (10)
W-s outparalogs: (1569) Bins: (0) (32) (94) (307) (403) (733)
B-s outparalogs: (1331) Bins: (7) (45) (87) (208) (333) (651)

Orthologs: (1316) Bins: (72) (725) (316) (132) (54) (17)
Inparalogs: (125) Bins: (13) (28) (48) (21) (8) (7)
W-s outparalogs: (1688) Bins: (124) (85) (123) (237) (415) (704)
B-s outparalogs: (1370) Bins: (6) (48) (129) (190) (394) (603)

Figure S6C

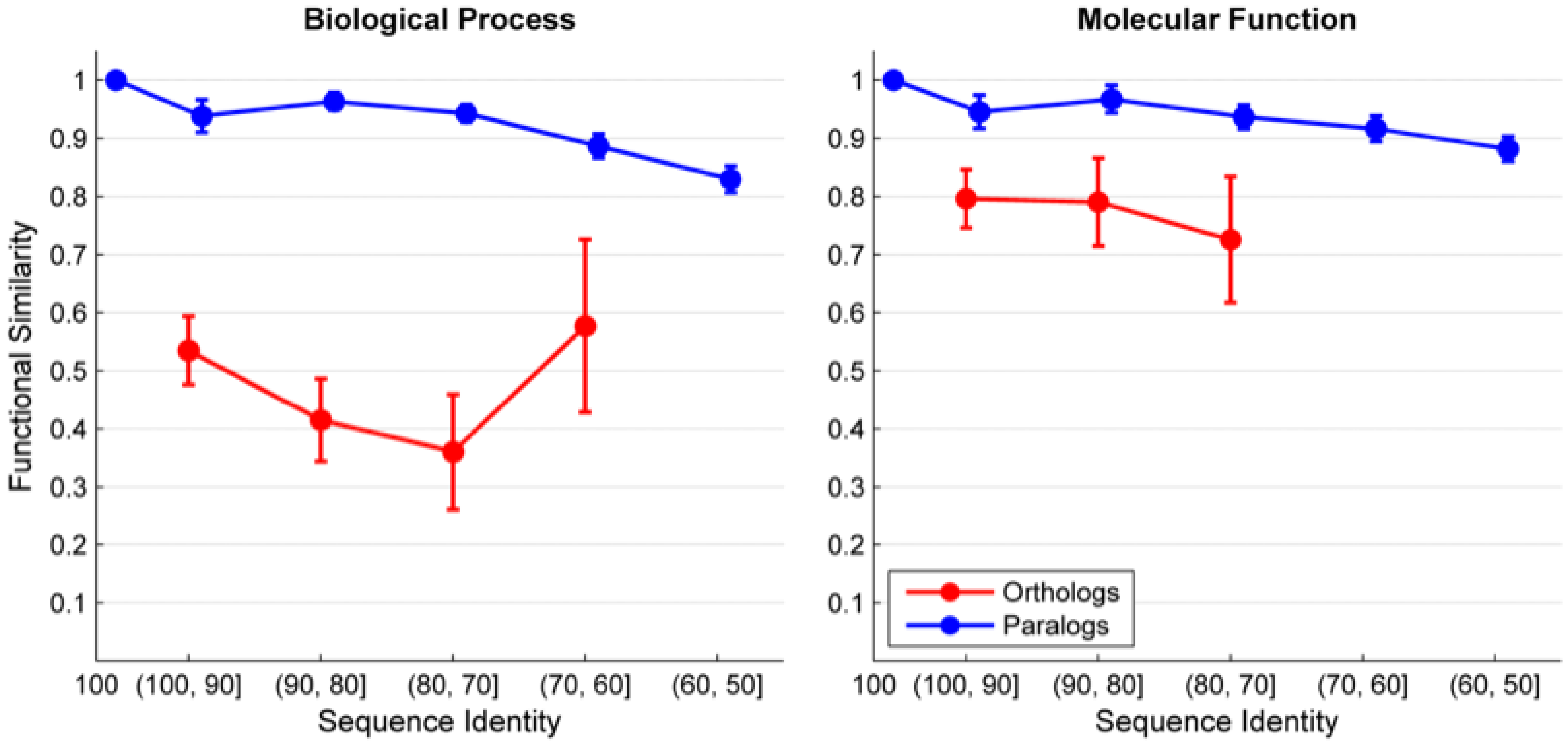

Orthologs: (91) Bins: (0) (42) (29) (12) (8) (0)
Paralogs: (768) Bins: (64) (45) (87) (201) (148) (223)

Orthologs: (67) Bins: (0) (38) (19) (10) (0) (0)
Paralogs: (649) Bins: (152) (49) (50) (99) (119) (180)

Figure S6D

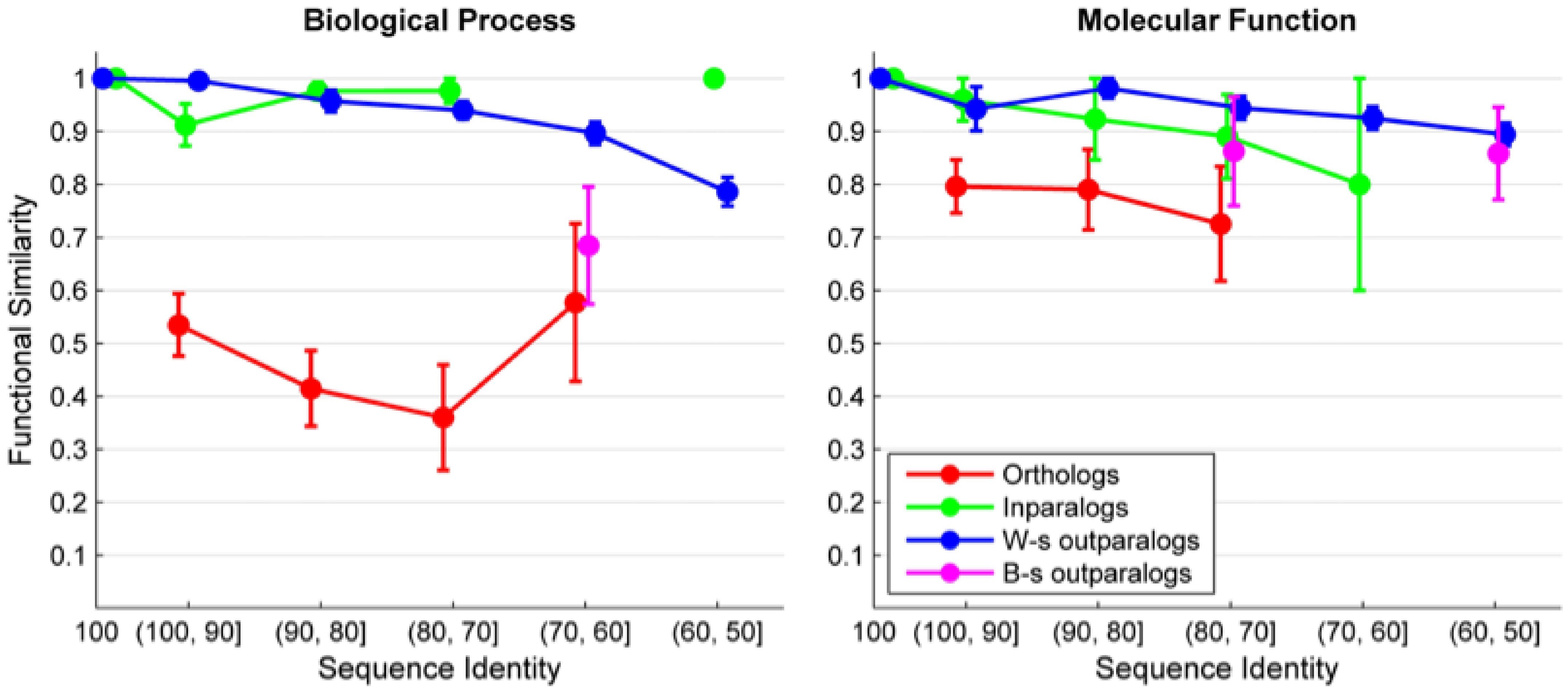

Orthologs: (91) Bins: (0) (42) (29) (12) (8) (0)
Inparalogs: (178) Bins: (55) (31) (28) (21) (0) (43)
W-s outparalogs: (576) Bins: (9) (13) (59) (177) (140) (178)
B-s outparalogs: (5) Bins: (0) (0) (0) (0) (5) (0)

Orthologs: (67) Bins: (0) (38) (19) (10) (0) (0)
Inparalogs: (103) Bins: (55) (25) (13) (5) (5) (0)
W-s outparalogs: (525) Bins: (97) (23) (35) (88) (112) (170)
B-s outparalogs: (13) Bins: (0) (0) (0) (6) (0) (7)
