# Supplementary material for: Testing the Ortholog Conjecture with Comparative Functional Genomic Data from Mammals
Source: PLoS Comput Biol. 2011 Jun 9;7(6):e1002073. doi: 10.1371/journal.pcbi.1002073 (PMC3111532; doi:10.1371/journal.pcbi.1002073)
Supplement: Figure S7 — The relationship between functional similarity and sequence identity using only protein pairs annotated with GO terms assigned by the same evidence code. All experimental (IDA, IEP, IGI, IMP, IPI), curator inferred (IC), and traceable author statement (TAS) evidence codes are included. A) human-mouse orthologs (red) and all paralogs (blue). B) human-mouse orthologs (red), inparalogs (green), within-species (W-s) outparalogs (blue), between-species (B-s) outparalogs (purple). Parts C) and D) show the same relationship using all experimental and curator inferred evidence codes, but excluding traceable author statements (TAS). Counts of gene pairs in each bin are listed below each figure. (PDF) [file pcbi.1002073.s007.pdf]

Figure S7A

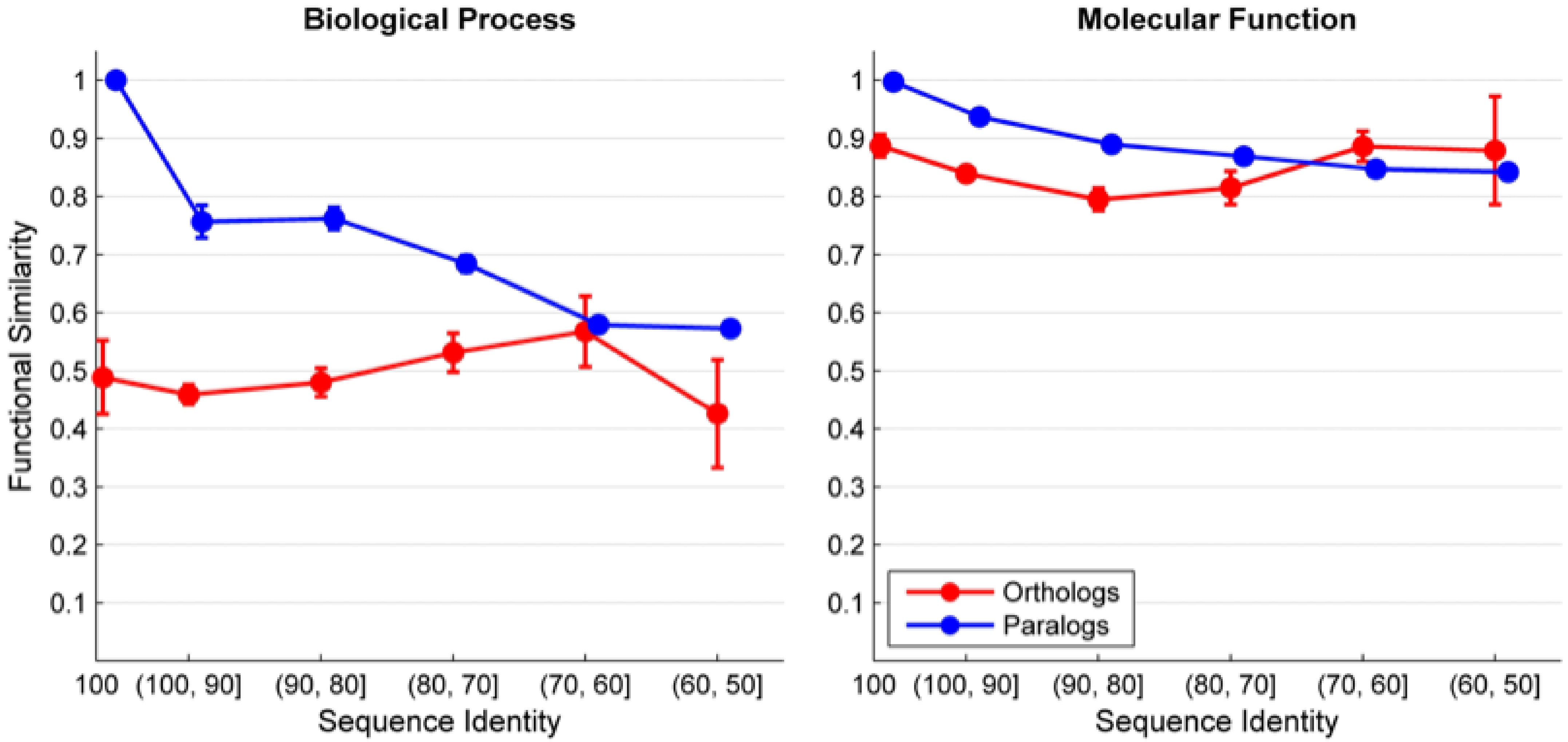

Orthologs: (704) Bins: (27) (362) (177) (87) (34) (17)
Paralogs: (3030) Bins: (98) (153) (261) (573) (741) (1204)

Orthologs: (1016) Bins: (76) (560) (236) (90) (43) (11)
Paralogs: (3625) Bins: (384) (231) (345) (543) (854) (1268)

Figure S7B

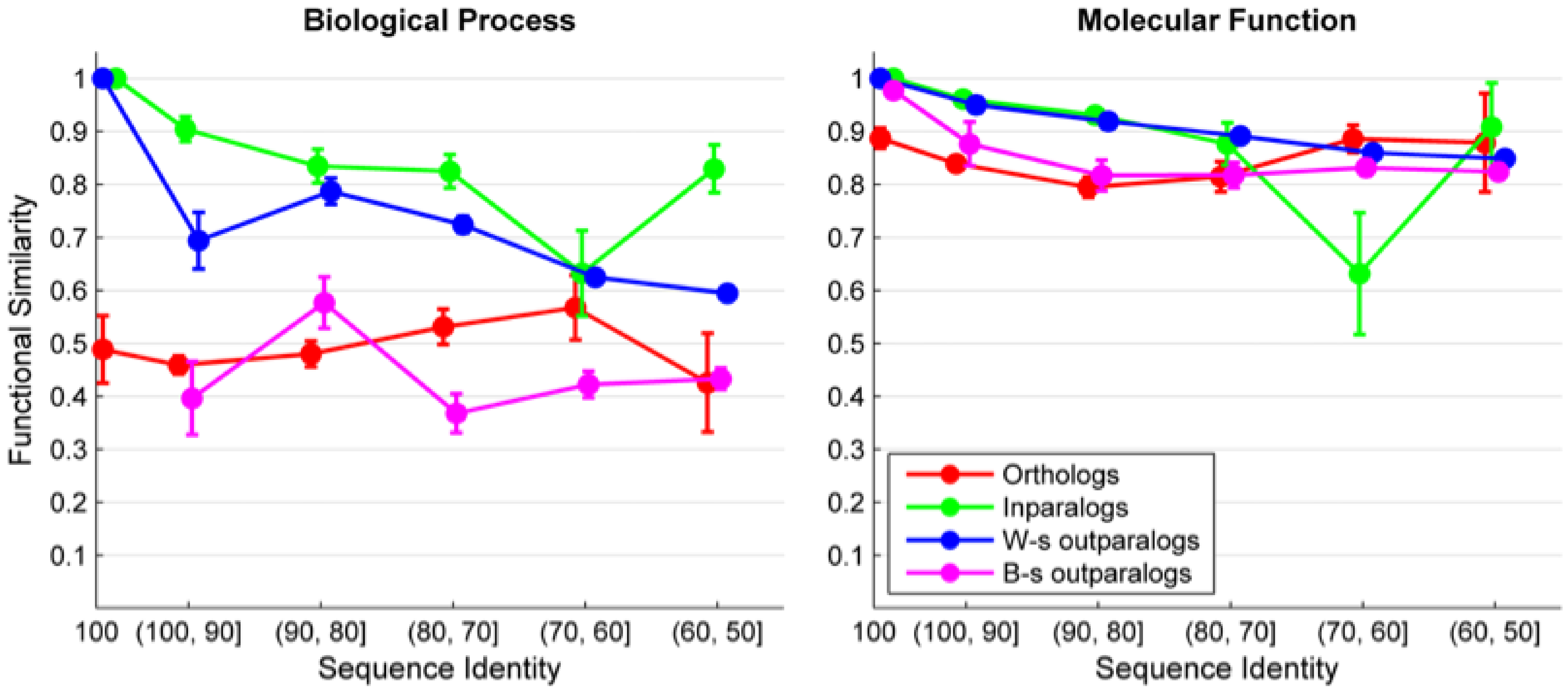

Orthologs: (704) Bins: (27) (362) (177) (87) (34) (17)
Inparalogs: (399) Bins: (86) (81) (74) (77) (21) (60)
W-s outparalogs: (2052) Bins: (10) (47) (139) (410) (552) (894)
B-s outparalogs: (577) Bins: (0) (25) (48) (86) (168) (250)

Orthologs: (1016) Bins: (76) (560) (236) (90) (43) (11)
Inparalogs: (311) Bins: (102) (75) (71) (36) (15) (12)
W-s outparalogs: (2286) Bins: (231) (104) (166) (350) (565) (870)
B-s outparalogs: (1028) Bins: (51) (52) (108) (157) (274) (386)

Figure S7C

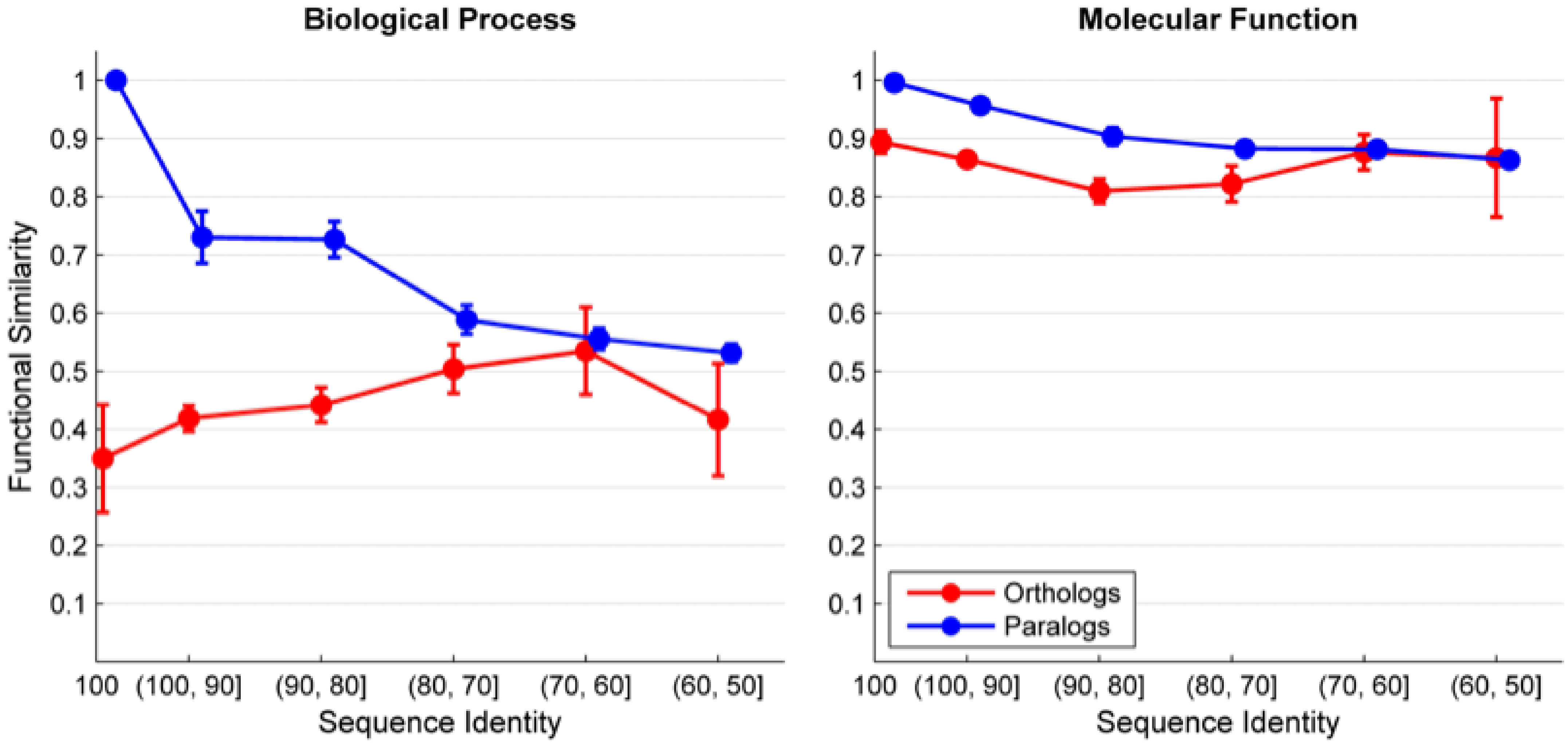

Orthologs: (414) Bins: (12) (198) (114) (55) (20) (15)
Paralogs: (1273) Bins: (53) (70) (105) (217) (337) (491)

Orthologs: (827) Bins: (65) (460) (188) (72) (32) (10)
Paralogs: (2196) Bins: (272) (174) (194) (337) (495) (724)

Figure S7D

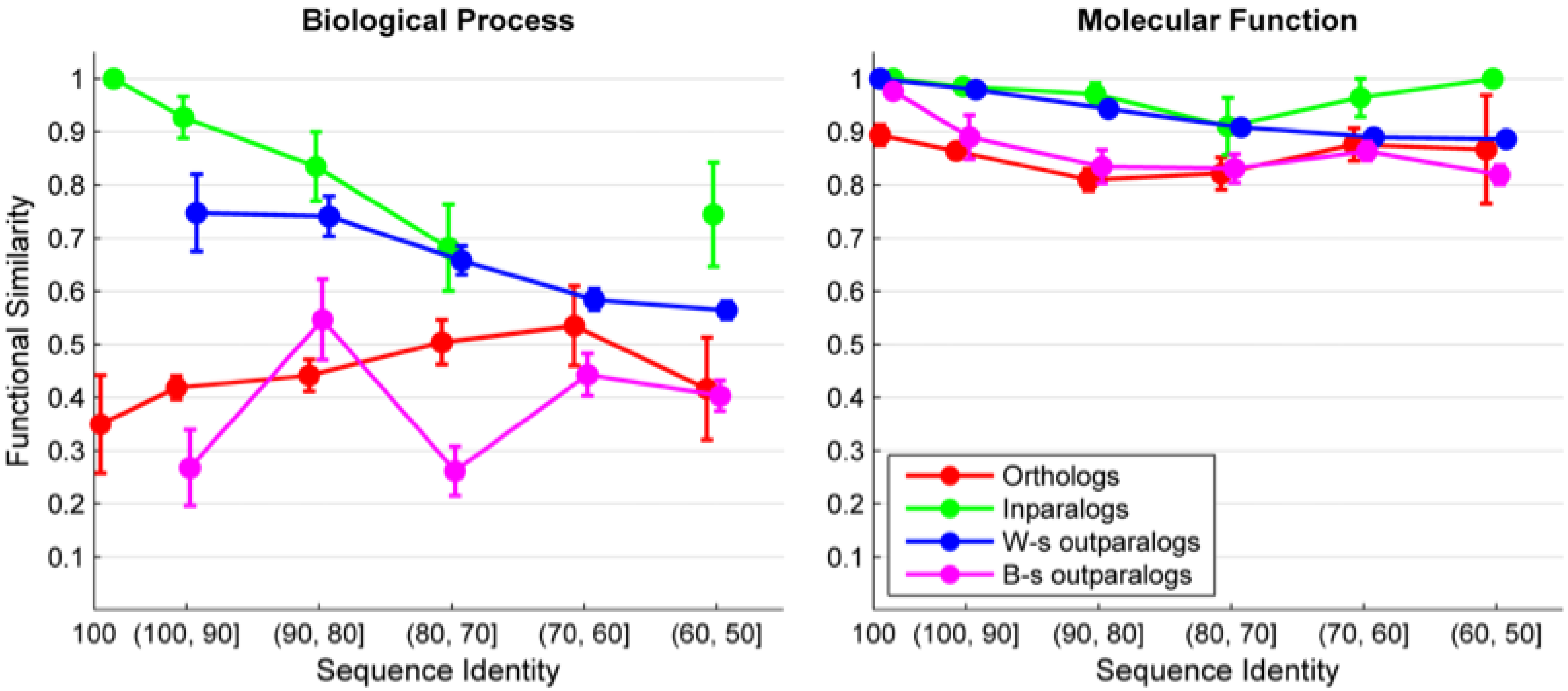

Orthologs: (414) Bins: (12) (198) (114) (55) (20) (15)
Inparalogs: (127) Bins: (47) (36) (21) (17) (0) (6)
W-s outparalogs: (886) Bins: (0) (18) (66) (161) (263) (378)
B-s outparalogs: (250) Bins: (0) (16) (18) (39) (70) (107)

Orthologs: (827) Bins: (65) (460) (188) (72) (32) (10)
Inparalogs: (163) Bins: (74) (36) (20) (19) (7) (7)
W-s outparalogs: (1302) Bins: (149) (91) (98) (204) (307) (453)
B-s outparalogs: (731) Bins: (49) (47) (76) (114) (181) (264)
