# Supplementary material for: Testing the Ortholog Conjecture with Comparative Functional Genomic Data from Mammals
Source: PLoS Comput Biol. 2011 Jun 9;7(6):e1002073. doi: 10.1371/journal.pcbi.1002073 (PMC3111532; doi:10.1371/journal.pcbi.1002073)
Supplement: Table S1 — Functional similarity within the nuclear receptor family in human and mouse. Of the total number of annotated proteins with both an ortholog and a paralog, the counts show the number in each category. Paralogs with higher functional similarity are further distinguished by whether the within-species or between-species outparalog was most similar. (DOC) [file pcbi.1002073.s009.doc]

**Supplementary Table 1.** Functional similarity within the nuclear receptor family in human and mouse. Of the total number of annotated proteins with both an ortholog and a paralog, the counts show the number in each category. Paralogs with higher functional similarity are further distinguished by whether the within-species or between-species outparalog was most similar.

| *Biological Process* | *Molecular Function* |
| --- | --- |
| Total annotated proteins: 40 | Total annotated proteins: 46 |
| Ortholog has higher functional similarity: 7 | Ortholog has higher functional similarity: 11 |
| Paralog has higher functional similarity: 33 | Paralog has higher functional similarity: 35 |
| Within-species outparalog: 22 | Within-species outparalog: 23 |
| Between-species outparalog: 11 | Between-species outparalog: 12 |
